# Spermidine-eIF5A axis is essential for muscle stem cell activation via translational control

**DOI:** 10.1038/s41421-024-00712-w

**Published:** 2024-09-10

**Authors:** Qianying Zhang, Wanhong Han, Rimao Wu, Shixian Deng, Jiemiao Meng, Yuanping Yang, Lili Li, Mingwei Sun, Heng Ai, Yingxi Chen, Qinyao Liu, Tian Gao, Xingchen Niu, Haixia Liu, Li Zhang, Dan Zhang, Meihong Chen, Pengbin Yin, Licheng Zhang, Peifu Tang, Dahai Zhu, Yong Zhang, Hu Li

**Affiliations:** 1 Bioland Laboratory (Guangzhou Regenerative Medicine and Health Guangdong Laboratory), Guangzhou, Guangdong China; 2 State Key Laboratory for Complex, Severe, and Rare Diseases, Institute of Basic Medical Sciences, Chinese Academy of Medical Sciences and School of Basic Medicine, Peking Union Medical College, Beijing, China; 3 Institute of Basic Medicine and Forensic Medicine, North Sichuan Medical College, Nanchong, Sichuan China; 4 Shandong University of Traditional Chinese Medicine, Jinan, Shandong China; 5 Senior Department of Orthopedics, The Fourth Medical Center of Chinese PLA General Hospital, Beijing, China; 6 National Clinical Research Center for Orthopedics, Sports Medicine & Rehabilitation, Beijing, China

**Keywords:** Muscle stem cells, Translation

## Abstract

Adult skeletal muscle stem cells, also known satellite cells (SCs), are quiescent and activate in response to injury. However, the activation mechanisms of quiescent SCs (QSCs) remain largely unknown. Here, we investigated the metabolic regulation of SC activation by identifying regulatory metabolites that promote SC activation. Using targeted metabolomics, we found that spermidine acts as a regulatory metabolite to promote SC activation and muscle regeneration in mice. Mechanistically, spermidine activates SCs via generating hypusinated eIF5A. Using SC-specific *eIF5A*-knockout (KO) and *Myod*-KO mice, we further found that eIF5A is required for spermidine-mediated SC activation by controlling MyoD translation. More significantly, depletion of eIF5A in SCs results in impaired muscle regeneration in mice. Together, the findings of our study define a novel mechanism that is essential for SC activation and acts via spermidine-eIF5A-mediated MyoD translation. Our findings suggest that the spermidine-eIF5A axis represents a promising pharmacological target in efforts to activate endogenous SCs for the treatment of muscular disease.

## Introduction

Adult stem cells often exist in a quiescent state and only re-enter the cell cycle to maintain tissue homeostasis or in response to tissue damage^1^. Skeletal muscle homeostasis during muscle regeneration and physiological aging tightly relies on resident muscle stem cells, which are also known satellite cells (SCs)^2,3^. In response to muscle injury, quiescent SCs (QSCs) activate rapidly, re-enter the cell cycle, and then differentiate and fuse to repair muscle^2,4–6^. During aging, there is a progressive decline of muscle mass and strength that is attributed to the defective activation and decreased self-renewal of SCs^7–9^. Therefore, understanding the molecular mechanisms of SC activation could suggest new avenues for treating muscle-degenerating diseases and counteracting muscle aging.

QSCs have a low metabolic rate and small size; they primarily comprise a nucleus surrounded by a thin layer of cytoplasm^10^. QSC activation requires the cellular metabolic state to be shifted to one that better matches the cells’ functional needs; this is termed “metabolic reprogramming”. The switch to glycolytic metabolism observed after QSC activation has been shown to downregulate the metabolite, NAD+ , which regulates QSC activation by directly influencing the epigenome^4^. Moreover, SCs exhibit restricted protein synthesis under quiescence, but undergo a high level of protein synthesis upon activation. However, we know little about the mechanism(s) by which SCs coordinate changes in metabolism and protein synthesis (translational control) to meet activation needs.

Polyamines are biogenic amines known to be involved in cell survival, cell proliferation, and protein synthesis^11^. Importantly, polyamines are also crucial precursors for the hypusination (a posttranslational modification) at a conserved lysine (K50) residue of eukaryotic translation initiation factor 5 A (eIF5A), which is required for all of its known functions as a translation factor^12,13^. Although originally thought to initiate translation, the hypusinated eIF5A (eIF5A^H^) actually promotes general translation elongation and supports the efficient translation of selective mRNA subsets, such as those containing consecutive proline codons^14–16^. Although polyamines have been reported to function in tumorigenesis and B cell activation^17^, we know little about the involvement of polyamine metabolism in controlling the cell fate of muscle stem cells.

Aberrant metabolic rewiring can have pathological consequences. Existing reports indicate that polyamine metabolism is reduced during aging and that, in human epidemiological studies, dietary polyamine uptake correlates with reduced cardiovascular and cancer-related mortality^18,19^. During aging, the progressive declines in muscle mass and strength are attributed to the defective activation and decreased self-renewal of SCs^3,9,20^. However, it is not yet known whether aging SCs can differ metabolically (e.g., in terms of polyamine metabolism) from their young counterparts, and whether this forms the basis for their impaired activation in aging.

Here, we report that spermidine acts as a regulatory metabolite to promote SC activation and muscle regeneration in mice via generating hypusinated eIF5A. Mechanistically, we reveal that eIF5A selectively translates *Myod* mRNAs during SC activation. MyoD has long been used as a molecular marker for SC activation^21,22^, but it was unknown whether MyoD is functionally required for SC activation. Here, we show that MyoD plays a pivotal role for SC activation via eIF5A-mediated translational control. Together, our findings not only suggest a novel role for the myogenic lineage-specific transcription factor, MyoD, during myogenic development, but may further provide a general mechanism by which eIF5A determines lineage specification and cell fate by selectively translating cell lineage-specific transcription factors (TFs) during development.

## Results

### Polyamine levels are significantly increased in activated SCs

**Fig. 1:**
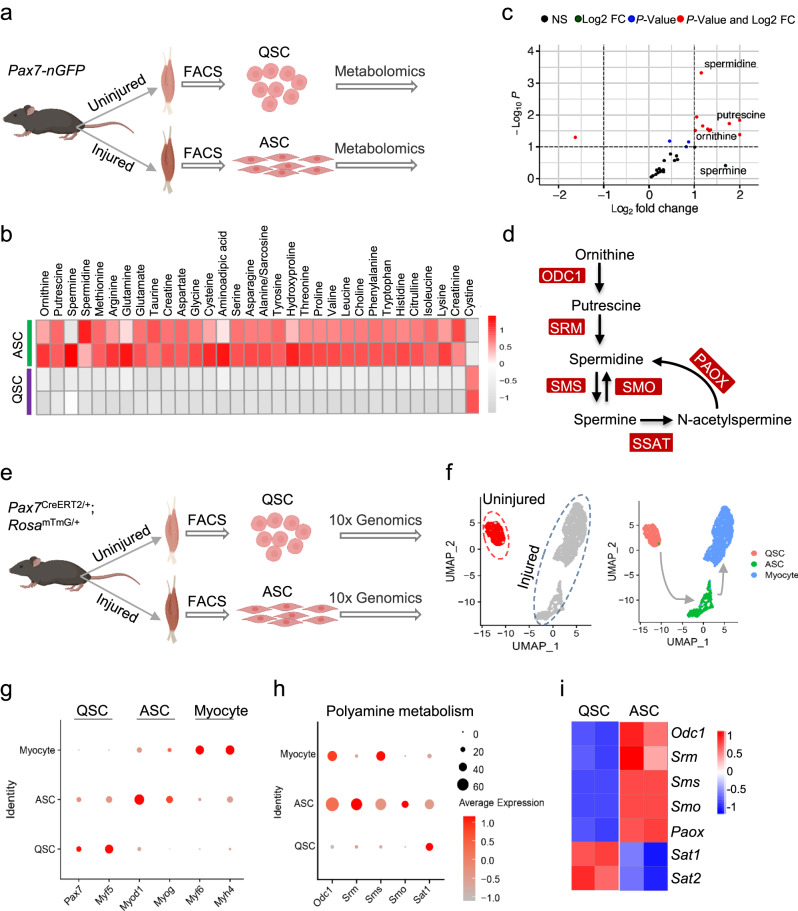
Polyamine levels are significantly increased in activated SCs. **a** Experimental scheme for analyzing metabolic alterations of quiescent satellite cells (QSCs) that were freshly FACS-isolated from uninjured *tibialis anterior* (TA) muscles of 8-week-old *Pax7-nGFP* mice, or activated satellite cells (ASCs) that were FACS-isolated from injured TA muscles on day 3 after cardiotoxin (CTX)-injection. Targeted metabolomics were used with three biological replicates in QSCs and five biological replicates in ASCs, each of which comprised a pool of SCs from 10 mice (for QSCs) or 3 mice (for ASCs). **b** Heatmap showing levels of amino acids and derivatives in ASCs and QSCs, as determined by targeted metabolomics. **c** Volcano plot displaying the most highly altered metabolites (red dots) in ASCs compared to QSCs, as assessed using a cutoff of |log_2_FC ≥ 1| and *P* < 0.05. **d** Schematic diagram showing the polyamine biosynthesis pathway, which involves six enzymes: ornithine decarboxylase (ODC1), spermidine synthase (SRM), spermine synthase (SMS), spermine oxidase (SMOX), spermidine/spermine N^1^-acetyltransferase (SSAT), and polyamine oxidase (PAOX). **e** Experimental scheme for scRNA-seq analysis of QSCs freshly FACS-isolated from uninjured TA muscles of 8-week-old *Pax7*^CreERT2/+^;*Rosa26*^mTmG/+^ mice, or ASCs FACS-isolated from injured TA muscles on day 1.5 after CTX injection. Each biological replicate of scRNA-seq comprises a pool of SCs from five mice (for QSCs or ASCs). **f** Uniform manifold approximation and projection (UMAP) plot showing distinct populations of QSCs and ASCs, as determined by the scRNA-seq described in **e**. **g** Bubble plot showing expression levels of marker genes for QSCs and ASCs, as determined by scRNA-seq. **h** Bubble plot showing the expression levels of polyamine biosynthesis-related genes in QSCs and ASCs, as determined by scRNA-seq. **i** Heatmap showing the expression levels of polyamine biosynthesis-related genes in QSCs and ASCs, as determined by bulk cell RNA-seq^4^.

QSC activation requires that the cellular metabolism shift to a state that better matches the cell’s functional needs; this shift is termed “metabolic reprogramming”. QSCs have a low metabolic rate, but their activation and entry into the cell cycle are characterized by major metabolic changes. To dissect the metabolic reprogramming that occurs during QSC activation, we performed targeted metabolomics analyses of QSCs and activated SCs (ASCs) (Fig. 1a). QSCs were freshly isolated by fluorescence-activated cell sorting (FACS) from the *tibialis anterior* (TA) muscles of 8-week-old *Pax7-nGFP* mice (Supplementary Fig. S1a). ASCs were FACS-sorted from CTX-injured TA muscles of 8-week-old *Pax7-nGFP* mice at 3 days post-injury (dpi) (Supplementary Fig. S1a). The FACS-resolved QSCs and ASCs were further characterized by examining the expression levels of the marker genes, *Pax7* and *Myod*, respectively (Supplementary Fig. S1b). Subsequently, amino acids and their derivatives in QSCs and ASCs were measured by ultra-performance liquid chromatography-tandem mass spectrometry (UPLC-MS/MS). The levels of 32 amino acids and derivatives showed differences between QSCs and ASCs (Fig. 1b). Nearly all of them were higher in ASCs than in QSCs (Fig. 1b), reflecting the urgent demand for amino acids to support the rapid increase of protein synthesis that occurs upon SC activation.

Interestingly, we found that amino acids and derivatives that are important in the polyamine biosynthesis pathway, including ornithine, putrescine, spermidine, and spermine, were increased in ASCs compared to QSCs (Fig. 1b and Supplementary Fig. S1c). Among them, spermidine was the most highly elevated metabolite in ASCs compared to QSCs (Fig. 1c). We quantified the absolute amount of spermidine and found that the level of spermidine was 2.25-fold higher in ASCs (19.2 μM) than in QSCs (8.5 μM) (Supplementary Fig. S1d). Polyamine biosynthesis in cells is regulated by six enzymes: ornithine decarboxylase (ODC1), spermidine synthase (SRM), spermine synthase (SMS), spermine oxidase (SMOX), spermidine/spermine N^1^-acetyltransferase (SSAT), and polyamine oxidase (PAOX) (Fig. 1d). To further explore the increased levels of polyamines in ASCs, we used single-cell RNA-seq (scRNA-seq) analysis to examine the expression levels of the genes encoding these enzymes in QSCs and ASCs (Fig. 1e and Supplementary Fig. S1e). Our combined analysis clearly demonstrated that three populations of cells, QSCs, ASCs and myocytes, revealed by the dimension reduction analysis with uniform manifold approximation and projection (UMAP) (Fig. 1f) and the heatmap showing differentially expressed genes (DEGs) among the three clusters (Supplementary Fig. S1f). Such three population of cells were validated by examining the levels of the marker genes, *Pax7* and *Myf5* in QSCs, *Myod* and *Myog* in ASCs, and *Myf6* and *Myh4* in myocytes (Fig. 1g). Consistent with the increased levels of polyamines in the ASCs, the expression levels of the genes encoding enzymes for polyamine biosynthesis, including *Odc1*, *Srm*, *Sms*, and *Smo*, were notably upregulated in ASCs compared to QSCs (Fig. 1h). In contrast, *Sat1*, encoding spermine N^1^-acetyltransferase, was significantly downregulated in ASCs compared to QSCs (Fig. 1h). Our observations were further corroborated by analyzing published bulk RNA-seq data from QSCs and ASCs (Fig. 1i and Supplementary Fig. S1g). Collectively, the results of our targeted metabolomic and scRNA-seq analyses show that polyamine levels are significantly increased in ASCs relative to QSCs.

### Blocking polyamine biosynthesis significantly suppresses SCs activation and skeletal muscle regeneration

**Fig. 2:**
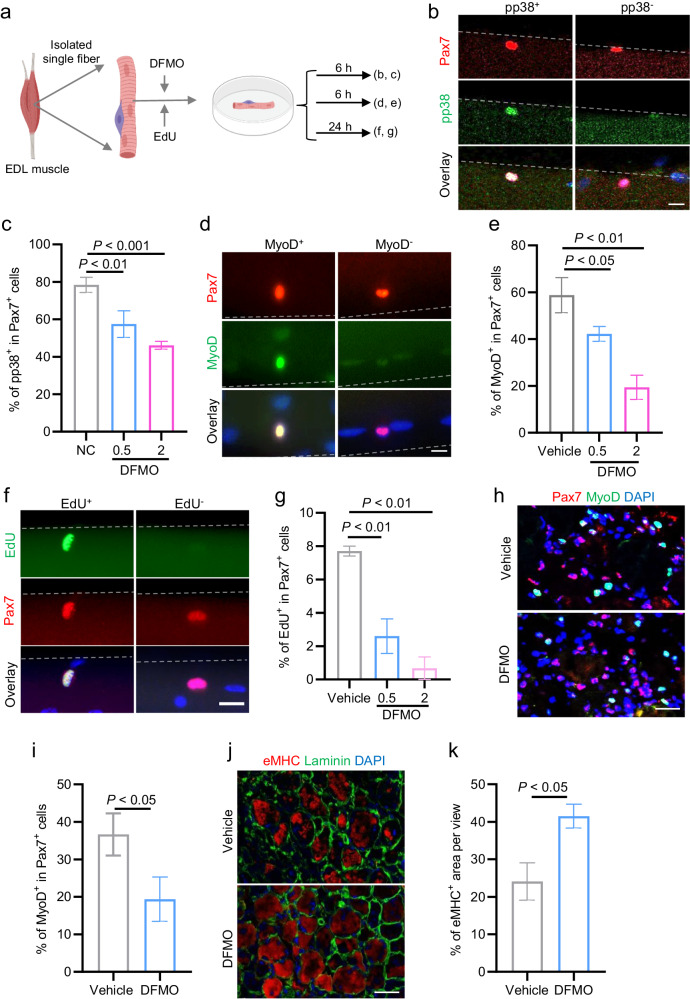
Blocking polyamine biosynthesis significantly suppresses SCs activation and skeletal muscle regeneration. **a** Schematic diagram showing the experimental strategy. Single myofibers isolated from *extensor digitorum longus* (EDL) muscles of C57BL/6j mice were treated with the ODC1 inhibitor, DFMO, for the indicated time points. SC activation was analyzed by immunostaining of phosphorylated form of p38, immunostaining of MyoD and Pax7, and EdU incorporation assay. **b** Representative images of immunofluorescent staining of phosphorylated p38 (pp38, green) and Pax7 (red) on explanted myofibers cultured for 6 h. DAPI (blue) served to visualize nuclei. Scale bar, 10 μm. **c** Percentage of pp38 positive (pp38^+^) cells among the total Pax7^+^ cells on explanted myofibers treated with the indicated dose of DFMO (0.5, 2 mM) for 6 h. Data are presented as mean ± s.e.m. from three independent experiments. One-way ANOVA. **d** Representative images of immunofluorescent staining of MyoD (green) and Pax7 (red) on explanted myofibers cultured for 6 h. DAPI (blue) served to visualize nuclei. Scale bar, 10 μm. **e** Percentage of MyoD^+^ cells among the total Pax7^+^ cells on explanted myofibers treated with the indicated dose of DFMO (0.5, 2 mM) for 6 h. Data are presented as mean ± s.e.m. from three independent experiments. One-way ANOVA. **f** Representative images of immunofluorescent staining of Pax7 (red) and EdU (green) on explanted myofibers cultured for 24 h. DAPI (blue) served to visualize nuclei. Scale bar, 10 μm. **g** Percentage of EdU^+^ cells among the total Pax7^+^ cells on explanted myofibers treated with the indicated dose of DFMO (0.5, 2 mM) for 24 h. Data are presented as mean ± s.e.m. from two independent experiments. One-way ANOVA. **h** Representative images of immunofluorescent staining for Pax7 (red) and MyoD (green) on cryosections of the injured TA muscle treated with DMFO or PBS as vehicle control at 1.5 days post injury (dpi). DAPI (blue) served to visualize nuclei. Scale bar, 50 μm. **i** Percentage of Pax7^+^/MyoD^+^ cells on the immunostained sections described in **h**. Mean ± s.e.m. *n* = 5 for each group. Two-tail Student’s *t*-test. **j** Representative images of immunofluorescent staining for embryonic form of myosin heavy chain (eMHC, red) and laminin (green) on cryosections of the injured TA muscle treated with DMFO or PBS as vehicle control at 5 dpi. DAPI (blue) served to visualize nuclei. Scale bar, 20 μm. **k** Percentage of eMHC^+^ cells on the immunostained sections described in **j**. Mean ± s.e.m. *n* = 5 for each group. Two-tail Student’s *t*-test.

Given the increased levels of polyamines observed during muscle stem cell activation, we examined the functional role of polyamine during SC activation and skeletal muscle regeneration. SC activation immediately triggers p38 mitogen-activated protein kinase (MAPK) signalling^23^ and MyoD translation^22,23^, which are generally used as early SC activation markers. EdU incorporation is also used to as later marker to monitor ASCs re-entry of cell cycle (G0 to S transition)^5,24–27^. Therefore, we first assessed how inhibition of polyamine biosynthesis affects SC activation by measuring the phosphorylated form of p38 (pp38) and the MyoD protein. The FACS-resolved SCs from *Pax7-nGFP* mice were treated with the ODC1 inhibitor, 2-difluoromethyl-ornithine (DFMO), for 6 h and subjected to western blotting analysis of levels of pp38 and MyoD protein. We observed that DFMO treatments significantly reduced protein levels of pp38 and MyoD (Supplementary Fig. S2a), suggesting that DMFO significantly suppresses SC activation. We further analyzed SC activation on ex vivo cultured single myofibers in presence of the inhibitor DFMO. The single myofibers isolated from the *extensor digitorum longus* (EDL) muscles of C57BL/6j mice were treated with DFMO for 6 h or 24 h (Fig. 2a), subsequently activation of SCs on explanted single myofibers was monitored by immunostaining of pp38 at 6 h (Fig. 2b, c), immunostaining of MyoD at 6 h (Fig. 2d, e), and the EdU incorporation assay at 24 h (Fig. 2f, g). Compared to the vehicle control, DFMO treatment significantly reduced the percentage of pp38^+^ cells (Fig. 2c), MyoD^+^ cells (Fig. 2e), and EdU^+^ cells (Fig. 2g), indicating that blocking polyamine biosynthesis significantly suppresses activation of SCs on ex vivo cultured single myofibers.

Next, we examined the in vivo function of polyamine in regulating SC activation and muscle regeneration. To this end, the TA muscle of C57BL/6j mice were injured with CTX and intramuscularly administrated with the inhibitor DFMO (10 μg) or PBS as a vehicle control. The SC activation was quantified by immunostaining of Pax7 and MyoD on cryosection of the injured-TA muscle at 1.5 dpi (Fig. 2h). Consistent with the results of our ex vivo experiments, DFMO treatment significantly reduced percentage of Pax7^+^/MyoD^+^ cells (Fig. 2i), indicating that blocking polyamine biosynthesis significantly suppresses SCs activation in vivo. Consequently, the muscle regeneration was compromised in the DFMO-treated mice compared to vehicle controls, evidenced by more embryonic form of myosin heavy chain positive (eMHC^+^) myofibers in DFMO-treated group at 5 dpi (Fig. 2j, k) and smaller size of the centralized regenerating myofibers at 7 dpi (Supplementary Fig. S2b–d). Together, these results show that blocking polyamine biosynthesis significantly suppresses SCs activation and muscle regeneration, indicating polyamine metabolism plays a pivotal role in regulating SC activation and skeletal muscle regeneration.

### Spermidine is required for SC activation and muscle regeneration

**Fig. 3:**
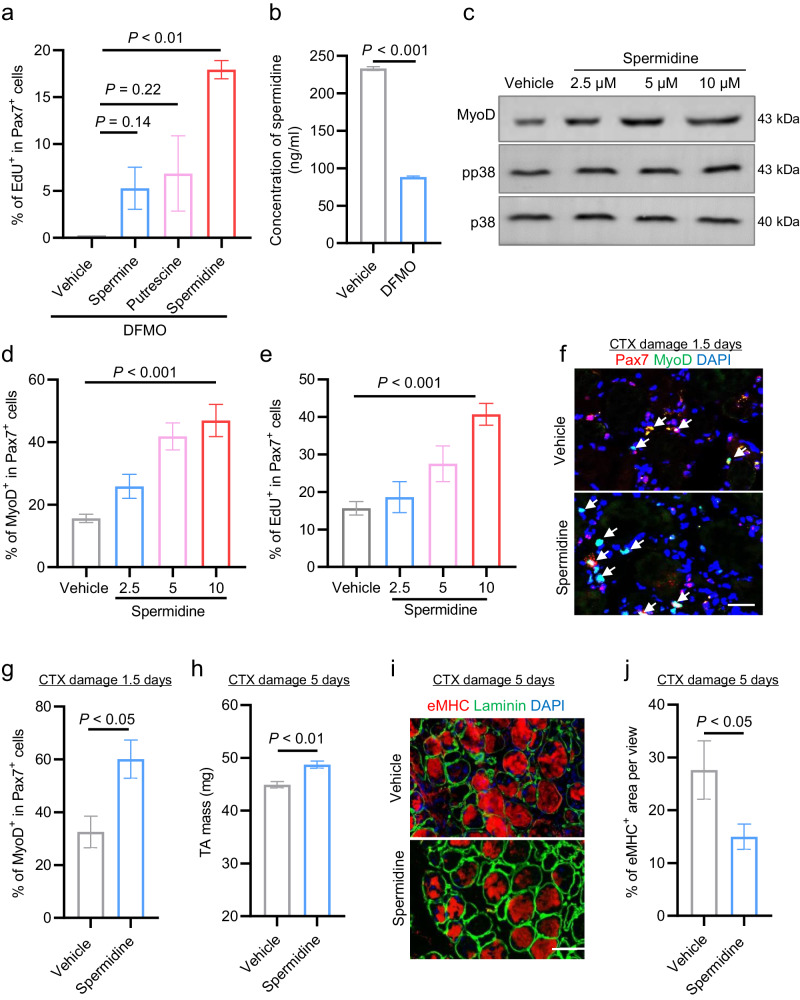
Spermidine is required for SC activation and muscle regeneration. **a** Percentage of EdU^+^ cells among total Pax7^+^ cells in single myofibers treated with 2 mM of DFMO and supplemented with spermine (10 μM), putrescine (10 μM), or spermidine (10 μM). *n* = 4 for each group. Mean ± s.e.m. One-way ANOVA. **b** Spermidine levels in FACS-sorted SCs cultured in presence of 0.5 mM of DFMO for 24 h, measured by LC-MS methods. *n* = 4 for each group. Mean ± s.e.m. One-way ANOVA. **c** Western blotting analysis showing protein levels of MyoD, phosphorylated p38 and total p38 in FACS-sorted SCs treated with the indicated dose of spermidine for 6 h. PBS treatment served as vehicle control. **d** Percentage of MyoD^+^ cells among total Pax7^+^ cells in EDL-derived single myofibers treated with various dose of spermidine (2.5, 5, 10 μM) for 3 h. *n* = 4 for each group. Mean ± s.e.m. One-way ANOVA. **e** Percentage of EdU^+^ cells among total Pax7^+^ cells in single myofibers treated with various doses of spermidine (0, 2.5, 5, 10 μM) for 24 h. *n* = 4 for each group. Mean ± s.e.m. One-way ANOVA. **f** Representative images of immunofluorescent staining for Pax7 (red) and MyoD (green) on cryosections of the injured TA muscle treated with spermidine or PBS as vehicle control at 1.5 dpi. DAPI (blue) served to visualize nuclei. Scale bar, 50 μm. **g** Percentage of Pax7^+^/MyoD^+^ cells on the immunostained sections described in **f**. Mean ± s.e.m. *n* = 5 for each group. Two-tail Student’s *t*-test. **h** Muscle mass of the injured TA treated with spermidine or PBS as vehicle control at 5 dpi. Mean ± s.e.m. *n* = 5 for each group. Two-tail Student’s *t*-test. **i** Representative images of immunofluorescent staining for eMHC (red) and laminin (green) on cryosections of the injured TA muscle treated with spermidine or PBS as vehicle control at 5 dpi. DAPI (blue) served to visualize nuclei. Scale bar, 20 μm. **j** Percentage of eMHC^+^ cells on the immunostained sections described in **i**. Mean ± s.e.m. *n* = 5 for each group. Two-tail Student’s *t*-test.

Polyamine species include putrescine, spermine and spermidine. To further examine which polyamine species contributed to DFMO-suppressed SC activation, we performed a rescue experiment by supplementing DFMO-treated single myofibers with spermidine, spermine, or putrescine. We observed that the DFMO-suppressed SCs activation was rescued by the exogenously added polyamines, with spermidine showing the greatest rescue efficacy (Fig. 3a), suggesting spermidine plays a critical role for SC activation. Putrescine had no overt effects on SC activation (Supplementary Fig. S3a, b). Although spermine promotes SC activation (Supplementary Fig. S3c), DFMO treatments actually only reduced spermidine levels (Fig. 3b) but no overt effects on spermine levels (Supplementary Fig. S3d). Together, those data suggested that DFMO-suppressed SC activation is attributed to depletion of spermidine.

Next, we further tested whether supplementation of spermidine promotes SC activation. To this end, the FACS-sorted SCs were treated with various dose of spermidine for 6 h and subjected to Western blotting assay for detection of pp38 and MyoD protein. Spermidine treatment significantly increased protein levels of pp38 and MyoD (Fig. 3c), suggesting that spermidine functionally promotes SC activation. We further verified the observation in ex vivo system. The EDL-derived single myofibers were treated with various dose of spermidine for 3 h (Fig. 3d) or 24 h (Fig. 3e), and SC activation was assessed by immunostaining of MyoD (Fig. 3d) or EdU incorporation assay (Fig. 3e), respectively. Compared to PBS treatment (vehicle), spermidine significantly and dose-dependently increased the percentage of MyoD^+^ cells (Fig. 3d) and EdU^+^ cells (Fig. 3e) among total Pax7^+^ SCs, indicating that spermidine acts as a regulatory metabolite in promoting SC activation.

Finally, we examined the in vivo function of spermidine during muscle regeneration by treating damaged TA muscles with 50 ng of spermidine. The SC activation was quantified by immunostaining of Pax7 and MyoD on cryosection of the injured-TA muscle at 1.5 dpi (Fig. 3f). Consistent with the results of our ex vivo experiments, spermidine treatment significantly increased percentage of Pax7^+^/MyoD^+^ cells (Fig. 3g), indicating that spermidine significantly promotes SCs activation in vivo. Consequently, the muscle regeneration was accelerated in the spermidine-treated mice compared to vehicle controls, evidenced by increased muscle mass (Fig. 3h), fewer eMHC^+^ myofibers in spermidine-treated group at 5 dpi (Fig. 3i, j) and greater size of the centralized regenerating myofibers at 7 dpi (Supplementary Fig. S3e, f). Altogether, these results reveal that spermidine acts as a regulatory metabolite in promoting SC activation and skeletal muscle regeneration.

### eIF5A is essential for SC activation and skeletal muscle regeneration

We next investigated the underlying molecular mechanism by which spermidine promotes SC activation. A known function of spermidine is to serve as a substrate for the hypusination of eukaryotic translation initiation factor 5A (eIF5A^H^) at a conserved lysine (K50) residue^16,28^. This metabolically regulated posttranslational modification is required for the activity of eIF5A in regulating protein synthesis from yeasts to human in vivo^12^. As protein synthesis is significantly enhanced during SC activation^11^, we speculated that SC activation might be cooperatively regulated by polyamine metabolism and protein synthesis through the spermidine-dependent hypusination of eIF5A.

**Fig. 4:**
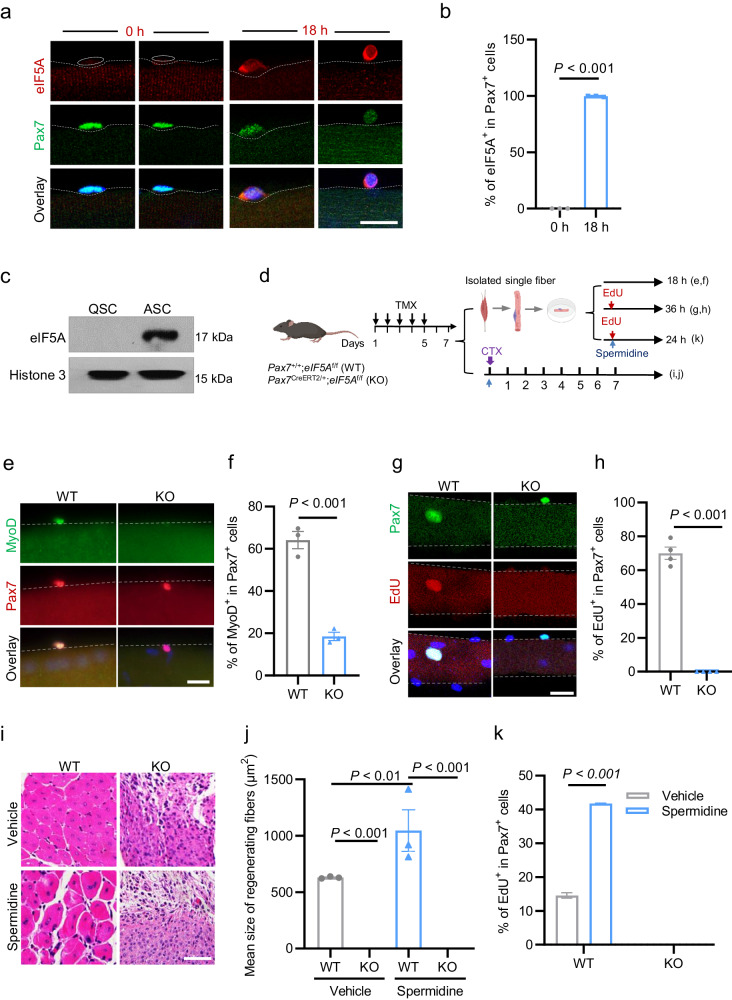
eIF5A is essential for SC activation and skeletal muscle regeneration. **a** Representative immunostaining of eIF5A (red) and Pax7 (green) in EDL-derived single myofibers from C57BL/6j mice that were cultured ex vivo for the indicated durations (0 h, 18 h). DAPI (blue) served to visualize nuclei. Scale bars, 20 μm. **b** Percentage of eIF5A^+^ cells among total Pax7^+^ cells in the single myofibers described in **a**. *n* = 3 per group. Mean ± s.e.m. Two-tail Student’s *t*-test. **c** Western blots showing protein levels of eIF5A in QSCs and ASCs. Histone 3 served as an equal loading control. **d** Experimental scheme for analyzing SC activation and CTX-induced muscle injury and regeneration using *Pax7*^CreERT2/+^;*eIF5A*^f/f^ (KO) and *Pax7*^+/+^;*eIF5A*^f/f^ (WT) control mice on day 2 after completion of a 5-day course of daily intraperitoneal tamoxifen (TMX) injections. **e** Representative immunostaining of Pax7 (red) and MyoD (green) in EDL-derived single myofibers obtained from KO and WT mice and cultured ex vivo for 18 h. DAPI (blue) served to visualize nuclei. Scale bars, 50 μm. **f** Percentage of MyoD^+^ cells among total Pax7^+^ cells in the single myofibers described in **e**. *n* = 4 per group. Mean ± s.e.m. Two-tail Student’s *t*-test. **g** Representative immunostaining of Pax7 (green) and EdU (red) in EDL-derived single myofibers obtained from KO and WT mice and cultured ex vivo for 36 h. DAPI (blue) served to visualize nuclei. Scale bars, 20 μm. **h** Percentage of EdU^+^ cells among total Pax7^+^ cells in the single myofibers described in **e**. *n* = 4 per group. Mean ± s.e.m. Two-tail Student’s *t*-test. **i** Representative H&E-stained cross-sections of TA muscles obtained from KO and WT mice on day 7 after combined treatment of CTX and spermidine or PBS control (vehicle). Scale bars, 100 μm. **j** Mean cross-sectional area of regenerated myofibers with centralized nuclei, measured from the H&E-stained cross-sections presented in **i**; *n* = 3 per group. Mean ± s.e.m. Two-way ANOVA. **k** Percentage of EdU^+^ cells among total Pax7^+^ cells in EDL-derived single myofibers obtained from KO and WT mice treated with 10 μM of spermidine for 24 h. PBS served as a control (vehicle). *n* = 4 for each group. Mean ± s.e.m. Two-way ANOVA.

To test this possibility, we first examined the expression pattern of eIF5A during SC activation. We found that mRNA levels of *eIF5A* in ASCs were 8-fold higher than those in QSCs (Supplementary Fig. S4a). Strikingly, all freshly isolated QSCs were eIF5A-negative when they were initially placed on EDL single myofibers (0 h) (Fig. 4a, b), and eIF5A-positive (eIF5A^+^) cells were increasingly detected in an activation timing-dependent manner thereafter (Fig. 4a). Of the total Pax7^+^ SCs, 100% were eIF5A^+^ after 18 h of ex vivo culture (Fig. 4b). Consistently, eIF5A protein was detected in FACS-sorted ASCs but not FACS-sorted QSCs (Fig. 4c). Overall, these data demonstrate that eIF5A is an activation-induced protein in ASCs.

To investigate the functional role of eIF5A in SC activation and skeletal muscle regeneration, we generated SC-specific *eIF5A*-knockout (KO) mice (*Pax7*^CreERT2/+^;*eIF5A*^f/f^) (Supplementary Fig. S4b) and used *Pax7*^CreERT2/+^ mice as the wild-type (WT) controls. Eight-week-old KO and WT control mice were intraperitoneally injected with tamoxifen (TMX). At 1 week after TMX induction, *eIF5A* knockout efficiency was measured at the mRNA level by real-time RT-PCR and at the protein level by immunofluorescent staining (Supplementary Fig. S4c, d). The results indicated that SC-specific *eIF5A* KO mice had been successfully established.

We then examined the effect of *eIF5A* deletion on SC activation by immunostaining of MyoD and EdU incorporation assays with SCs cultured on EDL-derived single fibers from KO and WT mice (Fig. 4d). We found that the percentage of MyoD^+^ cells among total Pax7^+^ cells on KO myofibers was significantly reduced (Fig. 4e, f). Remarkably, 70% of the total Pax7^+^ SCs grown on WT myofibers were EdU^+^, whereas all SCs grown on KO myofibers were EdU^–^ (Fig. 4g, h), indicating that eIF5A is essential for SC activation. SCs cultured on WT fibers proliferated and formed colonies after 72 h, whereas those cultured on *eIF5A*-KO fibers did not form colonies (Supplementary Fig. S4e, f), suggesting that *eIF5A* KO completely blocked SC activation rather than delaying it. The essential role of *eIF5A* in SC activation was further supported by our observation that SCs were smaller in cell size when cultured on myofibers from KO mice compared to WT controls (Supplementary Fig. S4d, g). The functional requirement of eIF5A was further evidenced by our observation that muscle regeneration was completely blocked in *eIF5A*-KO mice (Fig. 4i, j).

To test whether spermidine activates QSCs through eIF5A, the FACS-resolved SCs from KO and WT mice were treated with spermidine for 6 h and subjected to western blotting analysis. Protein levels of pp38 and MyoD were significantly elevated in spermidine-treated WT SCs, whereas spermidine lost its capability to activate p38 signaling and MyoD expression in SCs from *eIF5A* KO mice (Supplementary Fig. S4h), indicating that spermidine activates SCs dependent on eIF5A. We further corroborated the observation using ex vivo system. SCs cultured on EDL-derived single fibers from KO and WT mice were treated with spermidine for 24 h. EdU incorporation assays indicated that spermidine lost its capability to promote the activation of QSCs isolated from *eIF5A* KO mice (Fig. 4k). Consistently, spermidine was unable to enhance muscle regeneration in *eIF5A* KO mice (Fig. 4i, j). Collectively, these results provide ex vivo and in vivo evidence that spermidine and eIF5A play essential roles in SC activation and skeletal muscle regeneration.

### Spermidine stimulates SC activation by generating hypusinated eIF5A

**Fig. 5:**
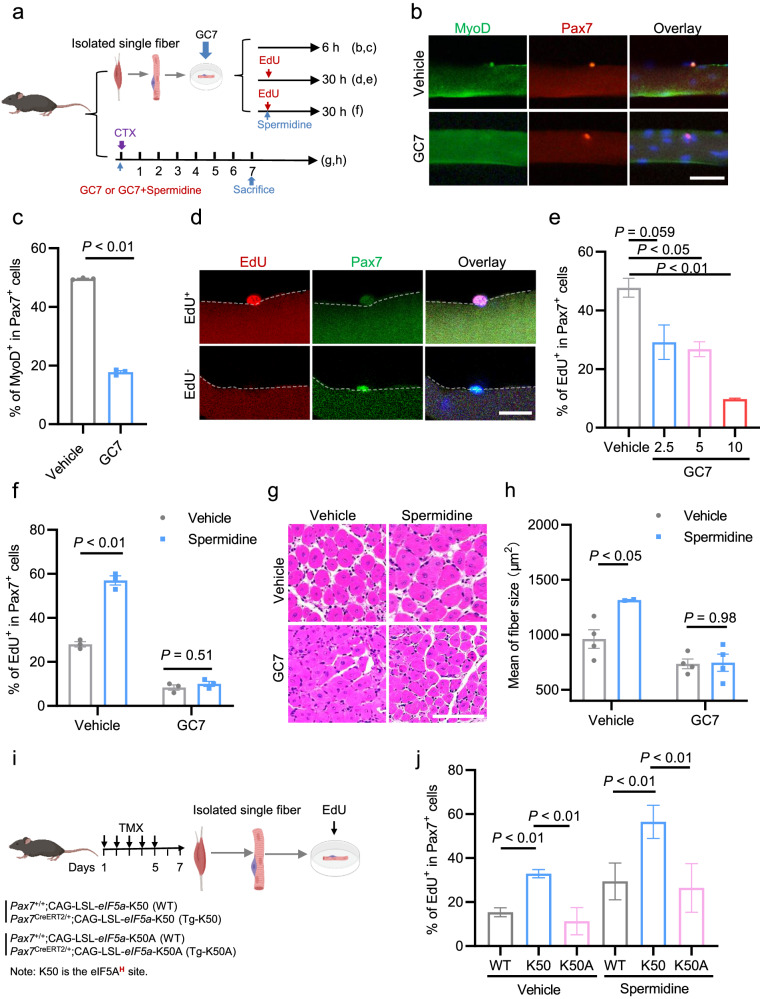
Spermidine stimulates SC activation by generating hypusinated eIF5A. **a** Experimental scheme for analyzing spermidine-mediated SC activation and muscle regeneration in the presence of GC7, an inhibitor of eIF5A hypusination. **b** Representative images of immunostaining for Pax7 (red) and MyoD (green) in EDL-derived single myofibers treated with 10 μM of GC7 for 6 h. DAPI (blue) served to visualize nuclei. Scale bar, 50 μm. **c** Percentage of Pax7^+^/MyoD^+^ cells described in **b**. *n* = 4 for each group. Mean ± s.e.m. Two-tail Student’s *t*-test. **d** Representative immunostaining of Pax7 (green) and EdU (red) in EDL-derived single myofibers obtained from C57BL/6j mice and cultured ex vivo for 30 h. DAPI (blue) served to visualize nuclei. Scale bar, 20 μm. **e** Percentage of EdU^+^ cells among total Pax7^+^ cells in EDL-derived single myofibers obtained from C57BL/6j mice and cultured ex vivo for 30 h with various doses of GC7 (2.5, 5, 10 μM). PBS served as a control (vehicle). *n* = 4 for each group. Mean ± s.e.m. One-way ANOVA. **f** Percentage of EdU^+^ cells among total Pax7^+^ cells in EDL-derived single myofibers obtained from C57BL/6j mice and treated with 10 μM of spermidine in the presence or absence of GC7 (10 μM) for 30 h. *n* = 3 per group. Mean ± s.e.m. Two-way ANOVA. **g** Representative H&E-stained cross-sections of TA muscles obtained from C57BL/6j mice on day 7 after CTX-induced injury and treated with spermidine (50 ng) in the presence or absence of the inhibitor, GC7 (50 μg). Scale bar, 100 μm. **h** Mean cross-sectional area of regenerating myofibers with centralized nuclei, as measured from the H&E-stained sections described in **g**. *n* = 4 per group. Mean ± s.e.m. Two-way ANOVA. **i** Experimental scheme for analyzing SC activation using *eIF5A*-transgenic (TG) mice (*Pax7*^CreERT2/+^;*CAG-LSL-eIF5A*-K50) and mutant form of *eIF5A* (K50A)-transgenic (mutTG) mice (*Pax7* ^CreERT2/+^;*CAG-LSL-eIF5A*-K50A). The corresponding wild-type (WT) controls were *CAG-LSL-eIF5A*-K50 and *CAG-LSL*-*eIF5A*-K50A mice, respectively. **j** Percentage of EdU^+^ cells among total Pax7^+^ cells in EDL-derived single myofibers obtained from WT, TG-K50, and mutTG-K50A mice treated with 10 μM of spermidine for 24 h. PBS served as a control (vehicle). *n* = 4 for each group. Mean ± s.e.m. Two-way ANOVA.

We next sought to understand how the spermidine-eIF5A axis activates QSCs at the molecular level. Given that the hypusination of eIF5A is required for its function^12^, we hypothesized that spermidine mediates QSC activation via hypusination of eIF5A. The hypusination of eIF5A can be inhibited by N1-guanyl-1,7-diaminoheptane (GC7), an inhibitor of deoxyhypusine synthase (DHPS), which is an essential enzyme for modifying a lysine residue to hypusine in eIF5A^16^. We tested our hypothesis by assessing how eIF5A hypusination blockade affected the SC activation. To this end, the FACS-resolved SCs were treated with various doses (2.5, 5 and 10 μM) of GC7 for 6 h and subjected to western blotting analysis. We observed GC7 dose-dependently reduced protein levels of pp38 and MyoD (Supplementary Fig. S5a). We also analyzed the SC activation on ex vivo cultured single fibers in presence of GC7 (Fig. 5a). GC7 treatment for 6 h significantly decreased percentage of MyoD^+^ cells among the total Pax7^+^ SCs on the explanted single myofibers (Fig. 5b, c). GC7 treatment for 30 h significantly and dose-dependently reduced the percentages of EdU^+^ cells among the total Pax7^+^ SCs in this system (Fig. 5d, e). Consistently, GC7 treatment for 72 h also remarkably reduced number of SC colonies grown on single myofibers (Supplementary Fig. S5b, c). To further verify that spermidine promotes SC activation through the hypusination of eIF5A, we treated SCs cultured on EDL-derived single myofibers with 10 μM of spermidine for 30 h in the presence or absence of GC7 (10 μM). Consistent with our earlier finding (Fig. 3e), spermidine treatment of this system significantly increased the percentage of EdU^+^ cells among the total Pax7^+^ SCs in the absence of GC7 (Fig. 5f), but GC7 treatment completely blocked the ability of spermidine to stimulate SC activation (Fig. 5f).

Next, we examined whether pharmacologically blocking the hypusination of eIF5A affects skeletal muscle regeneration in vivo. The TA muscles of 8-week-old C57BL/6j mice were injured with CTX and simultaneously injected with the hypusination inhibitor, GC7 (25 μg and 50 μg) (Supplementary Fig. S5d). Histological analysis at 7 dpi demonstrated that GC7 significantly and dose-dependently reduced the size of regenerating myofibers, which were characterized by centralized nuclei (Supplementary Fig. S5e). This observation showed that inhibition of eIF5A hypusination significantly impairs muscle regeneration in vivo (Fig. 5g, h). We then examined whether spermidine-enhanced muscle regeneration was dependent on eIF5A hypusination. To this end, the CTX-injured TA muscles were injected with either GC7 (50 μg) alone or a combination of GC7 (50 μg) and spermidine (50 ng). Again, blocking eIF5A hypusination with GC7 notably mitigated the ability of spermidine to stimulate skeletal muscle regeneration (Fig. 5g, h).

Finally, the requirement of hypusine-containing eIF5A protein for spermidine-stimulated SC activation was further confirmed by generating transgenic mice with TMX-inducible expression of either WT (CAG-LSL-*eIF5A*-K50, K50) or a loss-of-function mutant eIF5A (CAG-LSL-*eIF5A*-K50A, K50A) in which the hypusine-site lysine residue at position 50 was replaced by an alanine (A) residue (Supplementary Fig. S5f). To specifically express eIF5A in SCs, the mice were crossed with *Pax7*^CreERT2/+^ mice to generate K50 (*Pax7*^CreERT2/+^;CAG-LSL-*eIF5A*-K50) or K50A (*Pax7*^CreERT2/+^;CAG-LSL-*eIF5A*-K50A) mice. The SC-specific expression of WT or mutant eIF5A was induced in 8-week-old K50 and K50A mice intraperitoneally injected with TMX. Then expression of the transgenes of K50 and K50A in SCs of the transgenic mice were assessed by both RT-qPCR (Supplementary Fig. S5g–j) and immunostaining for eIF5A and Pax7 (Supplementary Fig. S5k). Consistent with the results presented in Fig. 4b, the endogenous eIF5A protein was only detected in ASCs of WT mice; in the TMX-treated K50 and K50A mice, however, 70%–80% of QSCs cultured on freshly isolated EDL single myofibers expressed eIF5A protein (Supplementary Fig. S5l). This indicated that the two lines of TG mice had been successfully established. We then asked whether overexpression of eIF5A in QSCs could promote SC activation in undamaged muscle (Fig. 5i). Our EdU incorporation assay showed that overexpression of K50-WT eIF5A promoted SC activation, as evidenced by increased percentages of EdU^+^ SCs compared to those in WT controls (Fig. 5j). However, expression of K50A-mutant eIF5A did not promote SC activation (Fig. 5j). Spermidine failed to increase the number of activated SCs cultured on single fibers isolated from K50A-mutant eIF5A mice (Fig. 5j). These findings further support the notion that hypusinated eIF5A is functionally required for QSC activation.

Taken together, our results obtained from pharmacological inhibition studies and genetic approaches provide compelling evidence indicating that spermidine stimulates SC activation and enhances muscle regeneration via the hypusination of eIF5A.

### Spermidine promotes activation of aged SCs and improves the muscle physiological function of aged mice

**Fig. 6:**
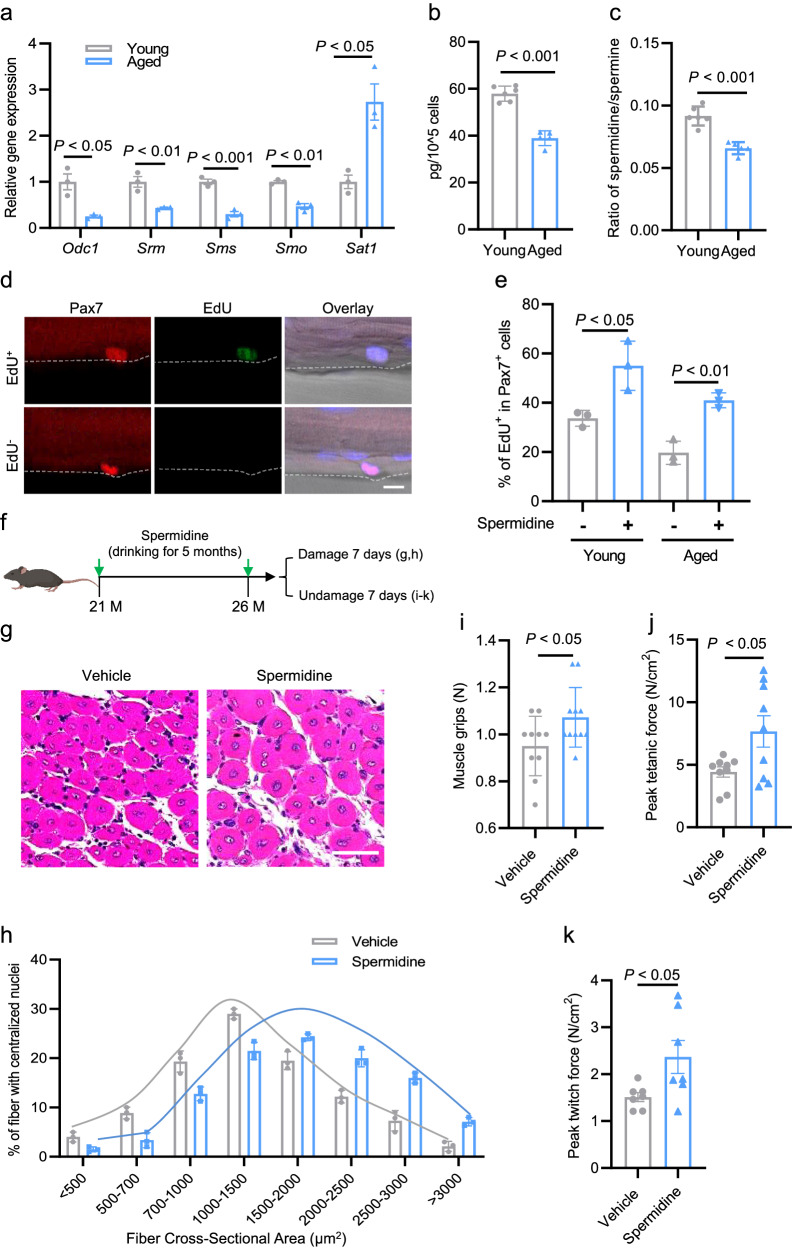
Spermidine promotes activation of aged SCs and improves muscle physiological function of aged mice. **a** Relative expression levels of genes encoding enzymes for polyamine biosynthesis in young and aged SCs, as determined by RT-qPCR. Mean ± s.e.m. Two-way ANOVA. **b**, **c** Level of spermidine (**b**) and the ratio of spermidine/spermine (**c**) in SCs isolated from young (6 months) and aged (30 months) mice. *n* = 5 for each group. Mean ± s.e.m. Two-tail Student’s *t*-test. **d** Representative images showing Pax7^+^ (red) and EdU^+^ (green) cells. DAPI (blue) served to visualize nuclei. Scale bar, 10 μm. **e** Percentage of EdU^+^ cells among total Pax7^+^ cells on single myofibers that were isolated from young and aged mice and treated with 10 μM of spermidine for 36 h. PBS served as a control (vehicle). Mean ± s.e.m. Two-way ANOVA. **f** Experimental scheme for exposure of aged mice (C57BL/6j) to 3 mM spermidine in the drinking water for 5 months, beginning at 21 months of age. **g** Representative images of H&E-stained cross-sections of CTX-injured TA muscle from the aged mice described in **f**. Scale bar, 50 μm. **h** Cross-sectional area of the regenerating myofibers characterized by centralized nuclei, described in **g**. *n* = 3 per group. Mean ± s.e.m. **i** Grip strength of the aged mice described in **f**. *n* = 10 per group. Mean ± s.e.m. Two-tail Student’s *t*-test. **j** Maximal tetanic force induced by in vitro electrical stimulation to elicit tetanic contractions in EDL muscles from the aged mice described in **f**. *n* = 9 per group. Mean ± s.e.m. Two-tail Student’s *t*-test. **k** Peak twitch force induced in EDL muscles of the aged mice described in **f**. *n* = 7 per group. Mean ± s.e.m. Two-tail Student’s *t*-test.

It has been reported that impaired activation of QSCs contributes to aging-associated physiological declines in skeletal muscle function. Based on our results showing that endogenous levels of spermidine are required for QSC activation in adult mice, we questioned whether the impaired activation of QSCs in aged mice could reflect aging-related decreases in the levels of endogenous spermidine in SCs. To test this possibility, we firstly examined the expression of genes encoding enzymes for polyamine biosynthesis, including *Odc1*, *Srm*, *Sms*, and *Smo* in SCs isolated from the aged mice. We found that the expression levels of these genes were significantly downregulated in the aged SCs (Fig. 6a). *Sat1*, encoding spermine N1-acetyltransferase, was significantly upregulated in aged SCs compared to young SCs (Fig. 6a), suggesting that polyamine biosynthesis pathways were significantly reduced in aged SCs compared to young SCs. In line with the downregulation of polyamine enzymes, spermidine levels and the ratio of spermidine to spermine were significantly decreased in aged SCs compared to young SCs (Fig. 6b, c and Supplementary Fig. S6a). Consistent with the decreased polyamine biosynthesis in the aged SCs, we also found that the EdU^+^ cell proportion was significantly reduced in aged mice compared to young mice (Fig. 6d, e and Supplementary Fig. S6b, c). Together, these results indicate that there is an association between reduced polyamine biosynthesis and impaired SC activation in aged mice.

Given that polyamine biosynthesis was significantly induced in ASCs upon activation and reduced in aged SCs, we hypothesized that the impaired activation of aged SCs might be due to the decreased levels of endogenous spermidine in aged mice. To test this possibility, aged QSCs isolated from 18-month-old mice were treated with spermidine (10 μM) for 36 h and activation was assessed by EdU incorporation assay. As found in young QSCs, spermidine treatment significantly increased the percentage of EdU^+^ cells in aged QSCs (Fig. 6d, e and Supplementary Fig. S6b, c).

Next, we investigated whether spermidine supplementation could improve the muscle physiological function of aged mice. To this end, we fed 21-month-old C57BL/6j male mice with spermidine (3 mM) via their drinking water for 5 months (Fig. 6f). Age-matched control animals received regular drinking water. The regeneration capacity and physiological performances of skeletal muscle in aged mice were assessed at the end of the spermidine treatment. We observed that spermidine treatment significantly recovered the muscle regeneration capacity in aged mice (Fig. 6g, h). Spermidine also significantly improved the muscle function of aged mice, as evidenced by elevated muscle grips (Fig. 6i) and enhanced muscle forces (Fig. 6j, k). The improved physiological function of the aged mice was also supported by reduced atrophy marker expression (Supplementary Fig. S6d–g). Collectively, our results indicate that spermidine supplementation contributes to maintaining muscle homeostasis and preventing muscle loss during aging in mice.

### eIF5A selectively translates the *Myod* mRNA in ASCs

**Fig. 7:**
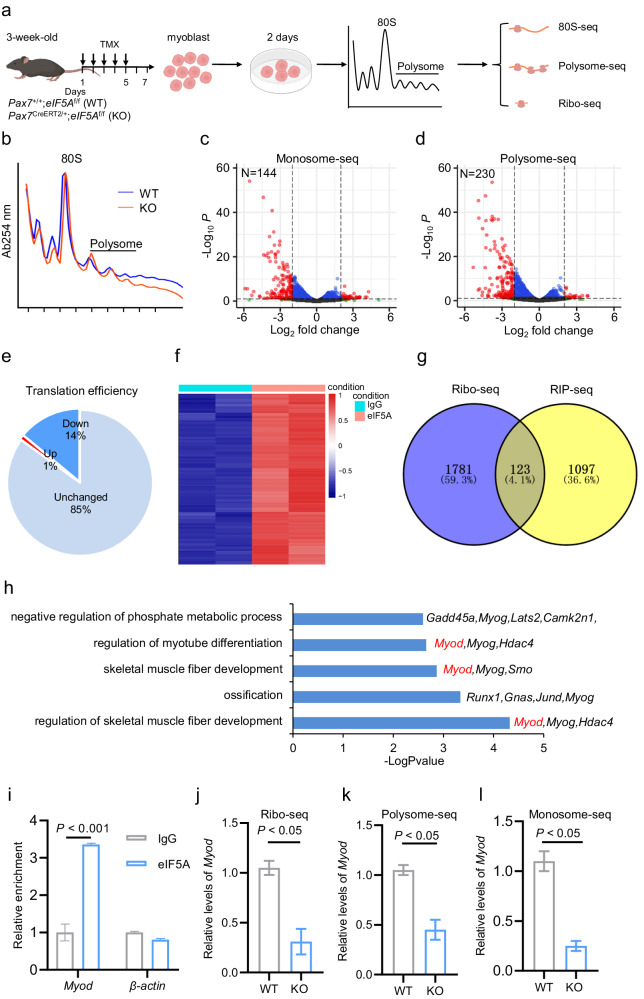
eIF5A selectively translates *Myod* mRNA in ASCs. **a** Schematic diagram showing monosome-seq, polysome-seq, and Ribo-seq analyses performed using primary myoblasts from 3-week-old *eIF5A*-KO mice and WT controls. **b** Profiling of monosomes and polysomes from *eIF5A*-KO (red) and WT control (blue) mice. **c** Volcano plot showing the differential mRNAs between *eIF5A*-KO and WT control mice, as determined by monosome-seq. **d** Volcano plot showing the differential mRNAs between *eIF5A*-KO and WT control mice, as determined by polysome-seq. **e** Pie chart displaying changes of translation efficiency (TE) in *eIF5A*-KO cells compared to WT controls. **f** Heatmap showing 1220 eIF5A-associated RNAs identified by RIP-seq. The RIP experiments were performed with eIF5A antibody using cell lysates of primary myoblasts from 2 to 3-week-old mice. **g** Venn diagram showing the number of overlapped mRNAs between Ribo-seq and RIP-seq. **h** Enriched GO terms of the overlapped mRNAs between Ribo-seq and RIP-seq. **i** Relative enrichment of *Myod* mRNA by RIP-seq analysis. Mean ± s.e.m. Two-way ANOVA. **j** Relative levels of *Myod* in *eIF5A*-KO and WT cells, as determined by duplicate experiments of Ribo-seq. Mean ± s.e.m. Two-tail Student’s *t*-test. **k** Relative levels of *Myod* in *eIF5A*-KO and WT cells, as determined by duplicate experiments of polysome-seq. Mean ± s.e.m. Two-tail Student’s *t*-test. **l** Relative levels of *Myod* in *eIF5A*-KO and WT cells, as determined by duplicate experiments of monosome-seq. Mean ± s.e.m. Two-tail Student’s *t*-test.

To elucidate the molecular mechanism underlying the function of eIF5A in regulating SC activation, we asked whether eIF5A exerts its function through mediating global or selective protein translation in ASCs. To this end, we first screened mRNAs known to be regulated by eIF5A in SCs by performing translational profiling via polysome-seq and monosome-seq analysis of polysome- and monosome-associated mRNAs in primary myoblasts isolated from 3-week-old TMX-induced *eIF5A*-KO mice and their WT littermates (Fig. 7a). Interestingly, the monosome and polysome profiles showed no overt difference between KO and WT cells, suggesting that *eIF5A* KO did not affect global translation in muscle cells (Fig. 7b). Further analysis demonstrated that, among the 21,032 mRNAs assessed by monosome-seq and polysome-seq, only 230 mRNAs had significantly fewer sequenced reads in *eIF5A* KO cells compared to WT controls, indicating that eIF5A selectively regulates the translation of a subset of mRNAs in muscle cells (Fig. 7c, d). To further corroborate this eIF5A-regulated selective translation in SCs, we performed Ribo-seq to calculate the translation efficiency (TE) of the cells used for monosome-seq and polysome-seq. We found that only 14% of mRNAs exhibited decreased translation efficiency in KO cells compared to WT controls (Fig. 7e). Together, these analyses reveal that eIF5A selectively translates a subset of mRNAs in muscle cells.

**Fig. 8:**
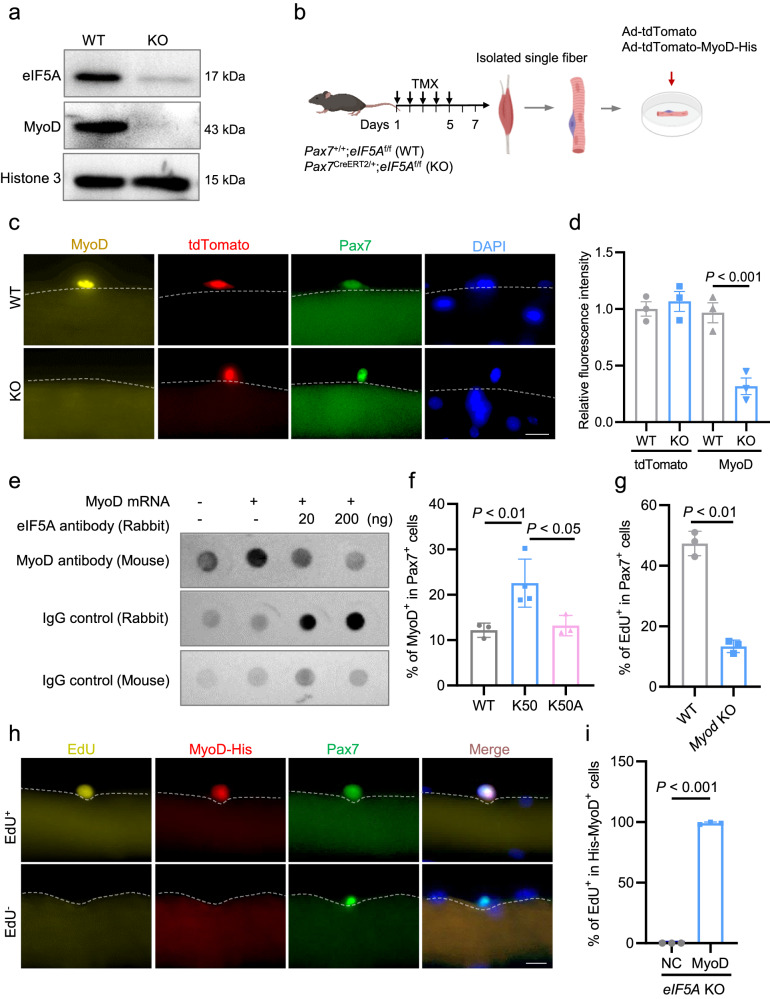
Spermidine-eIF5A-mediated translation of *Myod* is required for SC activation. **a** Western blotting analysis showing protein levels of MyoD and eIF5A in SCs from *eIF5A*-KO mice and WT controls. Histone 3 (H3) served as an equal loading control. **b** Schematic showing *eIF5A*-KO- or WT-derived single fibers infected with adenovirus encoding tdTomato and His-tagged-MyoD (Ad-tdTomato-MyoD-His), and cultured ex vivo for 24 h to induce SC activation. Adenovirus solely encoding tdTomato (Ad-tdTomato) served as control. **c** Representative images showing His^+^ (MyoD^+^, yellow), tdTomato^+^ (red), and Pax7^+^ (green) cells in the single myofibers described in **b**. DAPI served to visualize nuclei. Scale bar, 20 μm. **d** Quantified signal intensity of tdTomato and MyoD-His in the cells described in **b**, **c**. *n* = 3 per group. Mean ± s.e.m. Two-way ANOVA. **e** Representative dot blotting with antibody against MyoD, showing translation efficiency of full-length *Myod* mRNA (transcribed in vitro) in the eIF5A antibody-neutralized rabbit reticulocyte lysates and un-neutralized lysates as control. **f** Percentage of MyoD^+^ cells among total Pax7^+^ cells in EDL-derived single myofibers obtained from WT, K50, and K50A mice, and cultured ex vivo for 6 h. Mean ± s.e.m. One-way ANOVA. **g** Percentage of EdU^+^ cells among total Pax7^+^ cells in EDL-derived single myofibers obtained from *Myod*-KO mice and WT controls. *n* = 3 per group. Mean ± s.e.m. Two-tail Student’s *t*-test. **h** Representative images of EdU (yellow), MyoD-His (red), Pax7 (green) in the *eIF5A*-KO-derived single myofibers transfected with recombinant His-tagged MyoD and cultured ex vivo for 36 h. DAPI served to visualize nuclei. Scale bar, 20 μm. **i** Percentage of EdU^+^ cells among the total MyoD-His positive cells described in **h**. *n* = 3 per group. Mean ± s.e.m. Two-tail Student’s *t*-test.

To directly identify eIF5A-translated mRNAs, we performed immunoprecipitation with eIF5A antibody followed by RNA-seq (RIP-seq) using primary myoblasts from 2 to 3-week-old mice. The RIP-seq identified 1220 eIF5A-associated mRNAs (Fig. 7f). By overlapping the data from RIP-seq and Ribo-seq, we identified 123 mRNAs which might be more confident eIF5A-translated targets (Fig. 7g). The 123 mRNAs enriched GO terms of myotube differentiation and skeletal muscle fiber development (Fig. 7h). Notably, both *Myod* and *Myog* were in the gene list of those GO terms (Fig. 7h). Given MyoD is a well-documented early marker of SC activation, we focused on RIP-seq and Ribo-seq analysis on MyoD. The RIP-seq analysis revealed that *Myod* mRNA was enriched more than 3-fold in immunoprecipitants with eIF5A antibody compared to IgG controls (Fig. 7i). The Ribo-seq, polysome-seq and monosome-seq showed that the translation efficiency of *Myod* was significantly decreased in *eIF5A*-KO cells compared to WT controls (Fig. 7j–l). Consistent with The Ribo-seq data, western blotting analysis demonstrated that MyoD protein was only detected in WT cells but not in *eIF5A*-KO cells (Fig. 8a). Importantly, the *Myod* mRNA level did not differ between WT and *eIF5A*-KO cells (Supplementary Fig. S7). Together with the previous report that MyoD protein synthesis is translationally regulated during SC activation^22^, thus we proposed that eIF5A might mediate SC activation via controlling translation of MyoD protein.

To provide direct evidence validating that eIF5A mediates the translation efficiency of *Myod* mRNA in activated SCs, we expressed MyoD in activated SCs growing on single myofibers from *eIF5A*-KO and WT mice by infecting the cells with adenovirus encoding *His*-*Myod* and *tdTomato* and culturing them ex vivo for 24 h to induce SC activation (Fig. 8b). Expression of His-tagged-MyoD was visualized by immunostaining, and fluorescent tdTomato was detected as a control (Fig. 8c). We observed similar numbers of tdTomato^+^ SCs in the *eIF5A*-KO and WT controls, indicating that the infection efficiency and translation efficiency of *tdTomato* mRNA were similar in KO and WT cells (Fig. 8d). Consistent with the Ribo-seq data, all tdTomato^+^ SCs were strongly His^+^ (MyoD-His) in the WT group, whereas only a very weak MyoD-His signal was detected in tdTomato^+^ *eIF5A*-KO SCs (Fig. 8d). This appeared to further corroborate a requirement of eIF5A for translation of the *Myod* mRNA in the SCs. To provide direct evidence that MyoD is a target of eIF5A, we performed cell-free in vitro translation assay using rabbit reticulocyte lysates. To this end, eIF5A protein in the reticulocyte lysates was firstly neutralized with various dose of antibody against eIF5A, and then dot blotting with antibody against MyoD was performed to examine translation efficiency of full-length *Myod* mRNA transcribed in vitro in the eIF5A antibody-treated and untreated lysates. We found that *Myod* mRNAs were efficiently translated in the untreated lysates, however, the *Myod* translation was completely blocked in the lysates in which eIF5A was neutralized with its antibody (Fig. 8e). Together, our findings consistently reveal that MyoD is a bona fide target of eIF5A that selectively controls its translation efficiency in ASCs.

### Spermidine-eIF5A-mediated translation of MyoD is required for SC activation

As our earlier finding that eIF5A selectively translated *Myod*, we further analyzed whether eIF5A-mediated *Myod* translation depends on spermidine-mediated hypusination. Firstly, we demonstrated that spermidine treatment increased MyoD protein levels in activated SCs in vitro (Fig. 3c) and dose-dependently increased percentage of MyoD^+^ cells on ex vivo cultured single myofibers (Fig. 3d). Remarkably, blocking spermidine-mediated eIF5A hypusination with GC7 significantly reduced MyoD protein levels (Supplementary Fig. S5a) and decreased percentage of MyoD^+^ cells on ex vivo cultured single myofibers (Fig. 5b, c). Furthermore, the percentage of MyoD^+^ cells on single myofibers from K50–transgenic mice was significantly increased compared to that from WT littermates, however, there was no difference in percentage of MyoD^+^ cells between K50A-mutant–transgenic mice and WT controls (Fig. 8f). Together, we provide multiple lines of evidence to show that eIF5A-mediated *Myod* translation depends on spermidine-mediated hypusination.

It has been reported that *Myod* mRNA is present in both ASCs and QSCs, but translated only in ASCs^22,29,30^. Various mechanisms have been suggested to control MyoD translation during SC activation^22,31^, but no previous work had addressed whether MyoD protein is functionally required for SC activation. To approach the question, we generated inducible SC-specific *Myod*-KO mice (*Pax7*^CreERT2/+^;*Myod*^f/f^) (Supplementary Fig. S8a, b). SCs activation was evaluated by EdU incorporation of EDL-derived myofibers from the *Myod*-KO and WT littermates (Supplementary Fig. S8c). We found that the number of EdU^+^ cells was significantly reduced in *Myod*-KO compared to WT controls (Fig. 8g), indicating that MyoD is functionally required for SCs activation.

Given our findings that eIF5A is required for SC activation and selectively translates *Myod* in ASCs, we speculated that MyoD selectively translated by eIF5A may be functionally required for QSC activation. To test this possibility, we tried to rescue the activation defects of *eIF5A*-KO cells by delivery of recombinant His-tagged MyoD protein (MyoD-His) purified from bacterial cells (Supplementary Fig. S8d). To do that, we firstly examined the transcription activity of the bacterial produced recombinant MyoD protein in HEK293 cells by luciferase activity assay (Supplementary Fig. S8e–g). Then the recombinant MyoD protein was directly delivered into SCs on single myofibers from *eIF5A* KO mice, cultured ex vivo for 36 h and subjected to EdU incorporation assay (Fig. 8h). We found that almost 100% MyoD-His–positive cells were EdU^+^ detected in the *eIF5A*-KO SCs (Fig. 8i), indicating that recombinant MyoD could functionally rescue the activation defects of *eIF5A*-KO cells. Taken together, these results indicate that eIF5A-mediated selective translation of *Myod* is functionally required for QSC activation.

Collectively, our findings demonstrate that the spermidine-eIF5A-mediated translation of MyoD is required for SC activation.

## Discussion

We first report that polyamine biogenesis (metabolism) plays a regulatory role in SC activation, muscle regeneration, and muscle aging. We show that the endogenous levels of spermidine are significantly increased in activated SCs and that spermidine is functionally required for SC activation and muscle regeneration via its ability to generate hypusinated eIF5A. Moreover, we found that MyoD and the spermidine-eIF5A axis are essential for SC activation. Further mechanistic investigations revealed that the spermidine-eIF5A axis activates QSCs by selectively translating the *Myod* mRNA. Together, our findings reveal for the first time that a metabolic link between polyamine metabolism and MyoD protein synthesis plays an indispensable role in SC activation and muscle regeneration.

Although it has been appreciated that metabolic reprogramming occurs during QSC activation, the previous studies mainly focused on glycolysis and fatty acid oxidation^4^. Interestingly, some studies have indicated functional roles of polyamine metabolism in regulating SC behaviors. Using scRNA-seq, Machado et al. reported that polyamine metabolism induction during SC activation^5^. Garcia-Prat et al. reported that spermidine restores autophagy and prevent senescence of aged SC^32^. Chrisam et al. reported that systemic administration of spermidine reactivated autophagy, leading to a concurrent amelioration of the histological and ultrastructural muscle defects^33^. Zhang et al. reported that spermidine supplementation induced autophagy in SC and enhanced SC proliferation^34^. In this study, we performed experiments to examine whether spermidine mediates MuSCs activation via inducing autophagy by treating the MuSCs with autophagy inhibitor Chloroquine (CQ) (Supplementary Fig. S8h). We did not observe the effect of CQ on spermidine-mediated muscle stem cell activation, suggesting that spermidine-induced muscle stem cell activation might be independent of autophagy. Meanwhile, analysis of Ribo-seq data from SCs of *eIF5A KO* and WT mice did not show that eIF5A regulated autophagy associated proteins. Although eIF5A has been reported to regulate autophagy^17,35^, this regulation might be mediated through a cell-type-specific mechanism.

In addition, polyamine metabolism has been also investigated in other biological systems^19^ and spermidine, as a major polyamine, is known to play pleiotropic roles in regulating cellular functions. For example, Puleston et al. demonstrated that spermidine modulates mitochondrial respiration and macrophage activation^36^. In addition, spermidine was shown to regulate CD4^+^ T cell effector programs^37^. Puleston et al. reported that spermidine biosynthesis represents one of the most profound metabolic changes during T cell activation and is a central determinant of helper T cell lineage differentiation^38^. In the present study, we discovered that the metabolic pathway of polyamine biosynthesis is upregulated during muscle stem cell activation and the metabolite, spermidine, is functionally required for muscle stem cell activation. Our findings unveil an essential role of polyamine metabolism in controlling quiescent SC activation and provide the first evidence showing that spermidine can regulate the cell fate determination of muscle stem cells.

It has been reported that expression of genes encoding spermidine enzymes was regulated by different mechanisms. Most of the studies focused on regulation of gene encoding ornithine decarboxylase (ODC1), which is the enzyme catalyzing the first step of polyamine biosynthesis, and indirectly increasing the availability of spermidine for hypusine synthesis. For example, the MYC oncogene played a role in hypusine formation by driving ODC1 transcription^39,40^. Antizyme isoform 1 (Az1) downregulates the polyamine biosynthetic pathway by targeting ODC1 for proteolytic destruction via the 26 S proteasome^41^. The levels of ODC1 and polyamines are significantly reduced upon dCNBP depletion^42^. Meanwhile, D’Amico et al. also illustrated that Hedgehog-AMPK-CNBP axis regulate polyamine biosynthesis by mediating ODC1 protein levels^43^. Moreover, ODC1 expression and activity were also mediated by p53^44,45^ and PTEN–PI3K–mTOR complex 1 (mTORC1) pathway^46–48^ in various cancer cells. Interestingly, the pathways that regulate polyamine enzymes also play functional roles in SC activation. For example, AMPK pathway regulating polyamine synthesis amended activated muscle stem cell content in *mdx* muscle^49^. In response to tissue damage, Notch signaling pathway was downregulated and decreased p53 expression, and then activated polyamine biosynthesis and SC activation by regulating urea cycle^5,50^. In addition, previous study showed that mTORC1 activity was necessary and sufficient for the transition of SC from G_0_ into G_Alert_^51^. Although several signaling pathways have been implicated in SCs activation, there remains largely elusive for the regulatory signaling and mechanism underlying the induced expression of genes encoding polyamine enzymes during SCs activation. Addressing the question would be of great help to understand early activation of SCs in response to physiological or pathological stresses.

eIF5A is the only protein that is hypusinated by deoxyhypusine synthase (DHPS) and deoxyhypusine hydroxylase (DOHH) with spermidine as a substrate^12^. In the three decades since the discovery of eIF5A, several different functions have been attributed to this translation factor; most of the work to date has focused on its functions in cell proliferation and tumorigenesis^52^. For example, studies have shown that the neuron-specific ablation of eIF5A or DHPS leads to impairments in neurodevelopment and cognitive functions in mice^52^. eIF5A has been implicated in the neuronal differentiation and neurotrophic actions elicited by nerve growth factor^53^. Two studies performed using the eIF5A hypusination inhibitor, GC7, reported that eIF5A may function in regulating myogenic differentiation^15,54^. By generating inducible SC-specific *eIF5A*-KO mice, we herein found that eIF5A is required not only for the transition of SC cell status from quiescence to activation but, most importantly, for muscle regeneration in mice. Mechanistically, we further uncovered that spermidine activates SCs by generating functional eIF5A via hypusination. Together, our findings demonstrate that the spermidine-eIF5A axis plays an essential role in controlling the cell fate of muscle stem cells in vivo.

Translational control of protein synthesis is critical for cellular functions and disease development^55–57^. Interestingly, recent studies have provided some evidence supporting the existence of specialized ribosomes that preferentially translate different subsets of mRNAs in given cell types during development. Selective translation is being increasingly recognized as a major factor in determining the cellular functions for a subtype of proteins in a cell type-specific manner during development. Various models have been proposed for specialized ribosome-mediated selective translation^58^, but the underlying molecular mechanisms are still largely unknown. It has been documented that eIF5A functions globally in translation initiation, elongation, and termination^14,59^. However, one report showed that eIF5A reverses B cell senescence by regulating TFEB translation^17^. Here, we revealed that eIF5A selectively translates a subset of mRNAs that are functionally required for myogenic lineage specification and differentiation. These findings suggest that eIF5A may regulate selective translation by mediating the formation of specialized ribosomes in cells. Notably, eIF5A protein was not detected in QSCs, and its expression was highly induced upon SC activation. It is well known that MyoD transcripts exist in QSCs and are translationally controlled in SCs during activation^22,30^. Our findings suggest that the spatiotemporal expression of eIF5A may be a mechanism that drives the selective translation of the *Myod* mRNA by forming specialized ribosomes in activated SCs. This may reflect a general mechanism by which eIF5A determines lineage specification and cell fate by selectively translating cell lineage-specific TFs during development. Future studies are needed to validate this mechanism and its general significance in other cellular systems.

MyoD is a master TF for myogenic specification and differentiation^60,61^ and has long been used as a molecular marker for assessing SC activation^21,22^. However, no previous study had examined the functional requirement of MyoD for SC activation. Here, we not only uncover the essential role of MyoD in SC activation, we also mechanistically show that functional MyoD is orchestrated by eIF5A-mediated translational control. We recently have shown that MyoD metabolically regulates transcription of the myofiber-secreted granulocyte colony-stimulating factor (G-CSF) which acts as a metabolic niche factor required for establishing and maintaining the Pax7^Hi^ SC subpopulation in adult and physiological aged mice^62^. More recently, we also demonstrated that MyoD is a critical genome organizer in establishing the unique 3D genome architecture of muscle cells^63^. Here, we showed MyoD is functionally required for activation of QSCs. Together, our findings provide new evidence to expand our understanding of the previously unappreciated multi-functional roles of MyoD in myogenesis during development and regeneration.

Collectively, our results reveal that the spermidine-eIF5A axis links polyamine metabolism to translational control during muscle stem cell activation. The spermidine-eIF5A axis might therefore represent a pharmacological target that can be mobilized to counteract degenerative disease via activating endogenous adult stem cells.

## Materials and methods

### Mouse lines and animal care

All animal procedures were approved by the Animal Ethics Committee of Peking Union Medical College, Beijing (China). Mice were housed in a pathogen-free facility and had free access to water and standard rodent chow under the following conditions: 21 °C ambient temperature, 50–60% humidity, 12 h dark/light cycle. The floxed *eIf5A*, *eIF5A*-TG-K50, *eIF5A*-TG-K50A, and floxed *Myod* mice in the C57BL/6j background were generated by the Nanjing BioMedical Research Institute of Nanjing University (NBRI). The *Rosa26-mTmG* mice were kindly gifted by Dr. Shihuan Kuang (Purdue University). The *Pax7-CreERT2* mice were kindly gifted by Dr. Zhenguo Wu (The Hong Kong University of Science and Technology). The *Pax7-nGFP* Tg mice were kindly gifted by Dr. Shahragim Tajbakhsh (Institute Pasteur, France). The *Pax7-nGFP* mice used throughout this study were generated by crossing C57BL/6j mice (Vital River Laboratories Company, Beijing) with *Pax7-nGFP* Tg mice (C57BL6:SJL/J).

### FACS

Satellite cells from the skeletal muscles of *Pax7-nGFP* or *Pax7-CreERT2;Rosa26-mTmG* mice were fluorescently sorted as previously described^62^. Briefly, mononuclear muscle-derived cells were isolated from the hind-limb muscles of adult (2-month-old) mice by digesting the muscle chops with 2 U/mL dispase (Gibco, 17105041) and 0.2% collagenase (Gibco, 17101015) for 45 min. The cells were filtered through 70-µm and 40-µm cell strainers, suspended at 10^3^–10^7^ cells/mL, and directly sorted with a BD Aria II Cell Sorting System.

### Metabolomics analysis

The cells were thawed at 4 °C and mixed with 1 mL of cold methanol/acetonitrile/H_2_O (2:2:1, v/v/v). The homogenate was sonicated at low temperature (twice for 30 min per bout). The mixture was centrifuged for 20 min (14,000× *g*, 4 °C) and the supernatant was dried in a vacuum centrifuge. For LC-MS analysis, the samples were re-dissolved in 100 μL acetonitrile/water (1:1, v/v), adequately vortexed, and then centrifuged for 15 min (14,000× *g*, 4 °C). The supernatants were collected for LC-MS/MS analysis.

HPLC analyses were performed using an UHPLC (1290 Infinity LC, Agilent Technologies) coupled to a QTRAP (AB Sciex 5500). The mobile phase consisted of: A = 25 mM CH_3_COONH_4_ + 0.08% FA in water; and B = 0.1% FA in ACN. The samples were loaded to an automatic sampler at 4 °C, and the column temperature was maintained at 40 °C. A 2-µL aliquot of each sample was injected. The gradient was run at a flow rate of 250 μL/min, as follows: B was linearly reduced from 90% to 70% over 0 to 12 min, reduced to 50% over 12–18 min, reduced to 40% over 18–25 min, and then kept at 40% for 25–30 min. B was then increased to 90% over 30–30.1 min, and kept at 90% over 30.1–37 min. The QC samples used for testing and for evaluating the stability and repeatability of this system, at the same time, set the standard mixture of AA metabolites, used for correction of chromatographic retention time.

For MS/MS analysis (MRM) in ESI-positive modes, the conditions were set as follows: Source Temperature 500 °C, Ion Source Gas1 (Gas1) 40, Ion Source Gas2 (Gas2) 40, Curtain Gas (CUR) 30, Ion Spray Voltage Floating (ISVF) 5500 V, and MRM mode detection ion pair.

The Multiquant software was used to extract the chromatographic peak area and retention time. The AA standard-corrected retention time was used to identify the metabolites. The quality control samples were processed together with the biological samples. Detected metabolites in pooled samples with a coefficient of variation (CV) < 30% were denoted as reproducible measurements. To identify differential metabolites, statistical analyses between two sample groups were performed by calculating the fold changes and *P* values of metabolites. Student *t* test was used to obtain *P* values. Metabolites with *P* values < 0.05 and fold changes > 1.5 were taken as being significantly different between sample groups.

### scRNA-seq using the 10x Genomics Chromium Platform

scRNA-seq libraries were prepared with a Single Cell 3’ Reagent Kit as instructed by User Guide v2 (10x Genomics). Cell suspensions were loaded on a Chromium Controller instrument (10x Genomics) to generate single-cell gel bead-in emulsions (GEMs). GEM-reverse transcription (GEM-RT) was performed in a Veriti 96-well thermal cycler (Thermo Fisher Scientific). After RT, GEMs were harvested and the cDNAs were amplified and cleaned with an SPRIselect Reagent Kit (Beckman Coulter). Indexed sequencing libraries were constructed using a Chromium Single-Cell 3’ Library Kit (10x Genomics) for enzymatic fragmentation, end-repair, A-tailing, adaptor ligation, ligation cleanup, sample index PCR, and PCR cleanup. The libraries were sequenced with an Illumina NovaSeq 6000.

### Statistical analysis of scRNA-Seq data

We used Cell Ranger version 7.1.0 (10x Genomics) to process raw sequencing data and Seurat suite version 4.1.0 for downstream analysis. The Seurat R package was used for graph-based clustering and visualizations; all functions were enabled using this package or the standard R version 4.1.2 package and used with default parameters unless otherwise noted. Initially, we used Seurat to merge the two libraries and analyzed only cells (unique barcodes) that passed QC processing (above), expressed at least 500 genes, and expressed only genes found to be expressed in at least 3 cells. We also removed cells with > 1% mitochondrial genes. We applied library-size normalization to each cell using NormalizeData. The normalized expression for gene i in cell j was calculated by taking the natural log of the UMI counts for gene i in cell j divided by the total UMI counts in cell j multiplied by 10,000 and added to 1. To reduce the influence of variability on the number of UMIs and that of mitochondrial gene expression between cells on the clustering, we used the ScaleData function to linearly regress out these sources of variation before we scaled and centered the data for dimensionality reduction. Principle component analysis was run using RunPCA on variable genes identified using FindVariableGenes (*x* = (0.1,6), *y* = (0.5, 15)) and then extended to the full dataset with ProjectPCA. Based on the PCElbowPlot results, we decided to use 1 and 10 principal components (PCs) to cluster cells. We ran FindClusters to apply shared nearest neighbor (SNN) graph-based clustering to each sample (0.6).

### RNA-seq

Total RNA was extracted using TRIzol (Ambion, USA). The library construction was performed by Novagene. Sequencing libraries were generated using an NEBNext Ultra^TM^ RNA Library Prep Kit for Illumina (#E7530L, NEB, USA) following the manufacturer’s recommendations. Index codes were added to attribute sequences to each sample. Raw-sequencing data were mapped to the mouse genome mm10 assembly using HISAT with default parameters. DEGSeq2 was used to calculate the read coverage for each gene. Differentially expressed genes were filtered using a change greater than twofold and *P* value < 0.05 as a criterion for differential expression.

### Polysome profiling

The WT and eIF5A KO cells were treated with cycloheximide (CHX; 100 μg/mL) for 10 min, and then 5 × 10^6^ cells were lysed in lysis buffer (100 mM KCl, 2 mM MgCl_2_, 50 mM HEPES, 0.1% Triton X-100, and 10% glycerol, pH 7.4) supplemented with 20 U/mL RNase inhibitor (EDTA free), 100 μg/mL CHX, 1× cocktail (Roche), and 1 mM dithiothreitol (DTT). Cell lysates were centrifuged at 13,000× *g* at 4 °C for 15 min and the supernatant was loaded onto 20% to 50% sucrose gradients and ultracentrifuged with a SW41 rotor (Beckman) at 36,400 rpm/4 °C for 3 h. Gradients were fractionated by continually monitored A260 values using a BioComp Piston Gradient Fractionator equipped with a Bio-Rad Econo UV Monitor. Fractions representing the monosome peak and polysome peaks were pooled to produce a monosome sample and polysome sample, respectively. The isolated fractions were subjected to RNA extraction with TRIzol, and the extracted RNA samples were subjected to RNA-seq of monosome-seq and polysome-seq.

### Ribosome profiling

Cells were treated with CHX at 100 μg/mL for 1 min and collected, and 5 × 10^6^ cells were lysed in lysis buffer (5 mM MgCl_2_, 10% Triton X-100, Tris-HCl pH 7.5, and 150 mM NaCl) supplemented with 1 U/μL DNase I, 50 μg/mL CHX, and 100 mM dithiothreitol (DTT) for 30 min on ice. Cell lysates were centrifuged at 18,000× *g* at 4 °C for 15 min and the supernatant was digested with RNase I. Ribosome-protected RNA fragments (RPF) were obtained using MicroSpin S-400 column resin; the column enriched for ribosome-mRNA complexes and the targeted RNA fragments were purified by PAGE. The purified RNA fragments were end-repaired and A-tailed, and 5’ and 3’ joints were directly added to the ends. RNA reverse transcriptase was used to synthesize cDNA, and additional steps of PCR enrichment and PAGE-based screening of target fragments were used to obtain a library containing target fragments. The library was verified by quality control screening and sequencing.

The SE50 sequencing strategy was used for computer sequencing. The original sequencing data, contained in a Fastq file, were processed by removing low-quality reads (for which bases with mass value sQ ≤ 5 accounted for > 50% of the entire read), reads with more than 10% bases for which information could not be determined (designated as ‘N’), reads with 5’ joint contamination, reads lacking an inserted fragment and 3’ joint sequence, the 3’ joint sequence itself (which was required but then trimmed), rRNA reads, and tRNA reads. The Tophat2 software package (http://ccb.jhu.edu/software/tophat/index.shtml) was used to clean data from the mouse genome database (Ensembl Mus_musculus_Ensembl_97.gtf) for transcriptome comparison, and quantitative analysis of gene levels was performed using HTSeq. Since RPF are enriched for lengths around 30 nt (26–32 nt), the effect of experimental enrichment can be evaluated by statistical fragment segmentation.

### Single myofiber isolation and culture

Single myofibers were isolated from the EDL muscles of 2-month-old mice by digestion with collagenase I (Sigma, C-0130), as previously described^64^. Briefly, each muscle sample was incubated in 3 mL of 0.2% collagenase I in serum-free DMEM in a shaking water bath at 37 °C for 45–60 min. Digestion was considered complete when the muscle looked less defined and slightly swollen, with hair-like single fibers flowing away from the edges. The resulting myofibers were cultured in a Petri dish containing fiber medium for activation assay (DMEM supplemented with 20% FBS and 1% penicillin–streptomycin) or fiber medium for colony formation (DMEM supplemented with 20% FBS, 0.5% chick embryo extract, and 1% penicillin–streptomycin) in a 5% CO_2_ incubator at 37 °C for 24 h, 36 h, or 72 h.

### Immunofluorescence

The ex vivo cultured single fibers were fixed with 4% paraformaldehyde for 5 min, permeabilized in 0.1% Triton-X-100 in PBS for 15 min at room temperature, and then blocked with 3% bovine serum albumin for 30 min. Subsequently, the myofibers were incubated overnight at 4 °C with primary antibodies against eIF5A (Abcam, ab32407), Pax7 (DSHB, AB_528428), MyoD (Santa Cruz, SC-760) or His-Tag (Cell Signaling Technology, 9715). The myofibers were then washed with PBS containing 0.1% BSA and incubated for 2 h with fluorescein-conjugated secondary antibodies (Invitrogen, A32787, A55058 and A3254) and Hoechst or DAPI. After several washes with PBS, the myofibers were examined under a fluorescence microscope (Olympus). Five mice were assayed in each set of experiments.

For the EdU incorporation assay, single myofibers were cultured in fiber medium (DMEM supplemented with 20% FBS and 1% penicillin–streptomycin) supplemented with EdU (10 μM) for 24 h or 36 h. The fibers were then fixed in 4% PFA for 15 min and stained according to the provided instructions (Thermo Fisher Scientific, C10639).

### Recombinant MyoD purification

The PCR-amplified DNA fragment encoding full-length MyoD (residues 1-318) was cloned into a modified pET28a vector with an N-terminal His6-SUMO tag and Ulp1 protease site. The constructed expression vector, named pET28a-SUMO-MyoD-FL, was transformed into *Escherichia coli* strain BL21 (DE3) (Agilent Technologies, Santa Clara, CA, USA). The cells were grown in LB medium supplemented with 50 mg/mL kanamycin at 37 °C until the OD_600_ reached 0.6–0.8, then 0.2 mM isopropyl β-d-1-thiogalactopyranoside (J&K) was added to induce protein expression and the culture was continued overnight at 18 °C.

The cells were harvested by centrifugation at 5000 rpm (Thermo Fisher Scientific) for 15 min. The pellet was resuspended in buffer containing 20 mM Tris-HCl, pH 8.0, 500 mM NaCl, and 25 mM imidazole pH 8.0, the cells were lysed under high pressure using a JN-02C cell crusher, and the lysate was clarified by centrifugation at 17,000 rpm for 60 min at 4 °C (Beckman Coulter). Each supernatant was loaded onto a 5-mL HisTrap FF column (GE Healthcare, Beijing, China) pre-equilibrated with Buffer 1 (20 mM Tris-HCl, pH 8.0, 500 mM NaCl, and 25 mM imidazole pH 8.0). Recombinant His6-SUMO-MyoD-full-length (FL) was eluted from the column using elution buffer (Buffer 2, 20 mM Tris-HCl, pH 8.0, 500 mM NaCl, and 500 mM imidazole pH 8.0) with a stage-wise gradient on an ÄKTA™pure (GE Healthcare). His6-SUMO tags were cleaved by Ulp1 protease during dialysis against buffer S (20 mM Tris-HCl, pH 8.0 and 500 mM NaCl) and removed with a second-step HisTrap FF column (GE Healthcare). The MyoD-FL protein in the flow through was diluted with pre-cooled 20 mM Tris-HCl, pH 8.0 to ensure a low NaCl concentration, and excess nucleic acids were removed using a HiTrap SP FF column (GE Healthcare, Beijing, China) with buffer A containing 20 mM Tris-HCl, pH 8.0 and 200 mM NaCl and buffer B containing 20 mM Tris-HCl, pH 8.0 and 1 M NaCl. The eluted protein was concentrated by centrifugal ultrafiltration (Millipore Amicon Ultra, 10 K) and loaded onto a pre-equilibrated HiLoad Superdex 75 16/60 column (GE Healthcare) in buffer GF (20 mM Tris-HCl, pH 8.0, 500 mM NaCl, and 2 mM DTT) for final purification. All steps were performed on ice or at a low temperature.

### In vitro translation assay of MyoD

*Myod* mRNAs were in vitro transcribed using standard RNA Synthesis Kit (NEB S2040). MyoD protein was in vitro translated using the in vitro transcribed *Myod* mRNAs with Retic Lysate IVT kit (Thermo Fisher Scientific AM1200). For the eIF5A neutralization assay, the lysates were first incubated with the antibody against eIF5A at 4 °C for 5 min prior to translation assays. The in vitro translated MyoD proteins were detected by dot blotting using the antibody against MyoD (Santa Cruz, sc-377460).

### MyoD protein transfection

Single myofibers isolated from the EDL muscles of 2-month-old mice were seeded in 12-well plate. The purified recombinant MyoD protein (15 µg) were diluted in 20 mM HEPES buffer to bring the total volume up to 100 µL and then 4 µL of PULSin transfection reagent (Polyplus transfection) was added. The mixture was vortexed and left to incubate for 15 min at room temperature. During this incubation time, 900 μL pre-warmed medium (DMEM supplemented with 10% FBS) was added to each 12-well plate. The PULSin complexes were then added to the single myofibers and left to incubate for 3 h. Subsequently, the medium was discarded and 2 mL medium (DMEM supplemented with 20% FBS) with 10 μM EdU was added to the wells. After 36 h, EdU positive cells on single myofibers were analyzed.

HEK293 cells were seeded in 12-well plate. The purified recombinant MyoD protein were diluted in 20 mM HEPES buffer to bring the total volume up to 100 µL and then 4 µL of PULSin transfection reagent (Polyplus transfection) was added. The mixture was vortexed and left to incubate for 15 min at room temperature. During this incubation time, 900 μL pre-warmed medium without serum was added to each 12-well plate. The PULSin complexes were then added to the HEK293 cells and left to incubate for 3 h. Subsequently, the medium was discarded and 1 mL medium (DMEM supplemented with 10% FBS) was added to the wells.

### Luciferase reporter assay

To examine the transcription activity of the bacterial produced recombinant MyoD protein, the purified recombinant MyoD protein were transfected to HEK293 cells. After 12 h, the plasmid of luciferase reporter driven by a 575 bp basal myogenin promoter (GBBS) was used to transfect HEK293 cells, Empty pGL‐3 vector was used as a negative control, and co‐transfection with a Renilla luciferase plasmid (Promega) served as a transfection control. The results are expressed as the activity of firefly luciferase relative to that of Renilla luciferase.

### RNA immunoprecipitation

Cells were lysed with cell lysis buffer (Cell Signaling Technology) supplemented with protease inhibitor cocktail (Calbiochem, La Jolla, CA). Protein concentrations in extracts were measured using a bicinchoninic acid assay (Pierce). A volume of extract containing 200 mg protein was immunoprecipitated. For RNA immunoprecipitation assays, RNase Inhibitor (40 U/μL; TaKaRa) and protease inhibitor were added to the cell lysis buffer, and ribonucleoside–vanadyl complex (10 mM; New England BioLabs) was added to the wash buffer. Anti-eIF5A antibodies were obtained commercially. RNA was purified using phenol/chloroform extraction and ethanol precipitation.

### Western blotting analysis

The cells were lysed in a buffer containing 50 mM Tris pH 7.5, 150 mM NaCl, 0.5% Nonidet P40, and protease and phosphatase inhibitors. The cell lysates were clarified by centrifugation at 12,000× *g* for 10 min. Total proteins (10 µg) were resolved by SDS-PAGE, transferred to a polyvinylidene fluoride membrane, and immunoblotted overnight at 4 °C with primary antibodies against eIF5A (Abcam, ab32407, 1:500), MyoD (Santa Cruz, SC-760, 1:500), and histone 3 (Cell Signaling Technology, 9715, 1:1000). The blots were washed with 1× TBST washing buffer for 30 min, incubated with horseradish peroxidase-conjugated secondary antibodies (Zhongshanjinqiao Corporation) for 1 h at room temperature, and washed with 1× TBST washing buffer for 30 min. Each blot was placed into Detection Solution (Thermo Fisher Scientific, 34580), incubated for 1 min at room temperature, and exposed to X-ray film.

### RNA extraction and RT-qPCR

Total RNA was extracted from skeletal muscles using the TRIzol reagent (Invitrogen) and reverse transcribed with reverse transcriptase (Fermentas). Real-time quantitative PCR analyses were performed in triplicate using the Fast Eva Green qPCR Master Mix (Bio-Rad). *β-actin* was used as an internal control for RT-qPCR analyses.

### Muscle injury and regeneration

Muscle regeneration was induced by injections of CTX (Shanghaiboyao, ZX0005). Mice were anesthetized by intraperitoneal injection of ketamine (10 mg/kg) and xylazine (1 mg/kg). For monitoring of muscle regeneration, muscle injury was induced in 8-week-old mice by injecting CTX (50 μL of 10 μM CTX in phosphate-buffered saline, PBS) into the mid-belly of the right TA muscle. As an internal control, the left TA muscle of each mouse was injected with PBS (50 μL). Muscles were harvested 7 days after injection to assess the completion of regeneration and repair.

### Statistical analysis

Data are presented as means ± s.e.m. For statistical comparisons of two conditions, the two-tail Student’s *t*-test was used. Comparisons of multiple groups were made using one- or two-way ANOVA. All experiments were repeated at least three times, and representative experiments are shown. Statistical analysis was performed using GraphPad Prism.

## Supplementary information

Supplementary Information

## Data Availability

The raw sequence data reported in this paper have been deposited in the Gene Expression Omnibus (GEO) with accession number: GSE221643.
