# Supplementary material for: Spermidine-eIF5A axis is essential for muscle stem cell activation via translational control
Source: Cell Discov. 2024 Sep 10;10:94. doi: 10.1038/s41421-024-00712-w (PMC11383958; doi:10.1038/s41421-024-00712-w)
Supplement: Supplementary file 1 — Supplementary Information [file 41421_2024_712_MOESM1_ESM.pdf]

Supplementary Figures and Figure legends

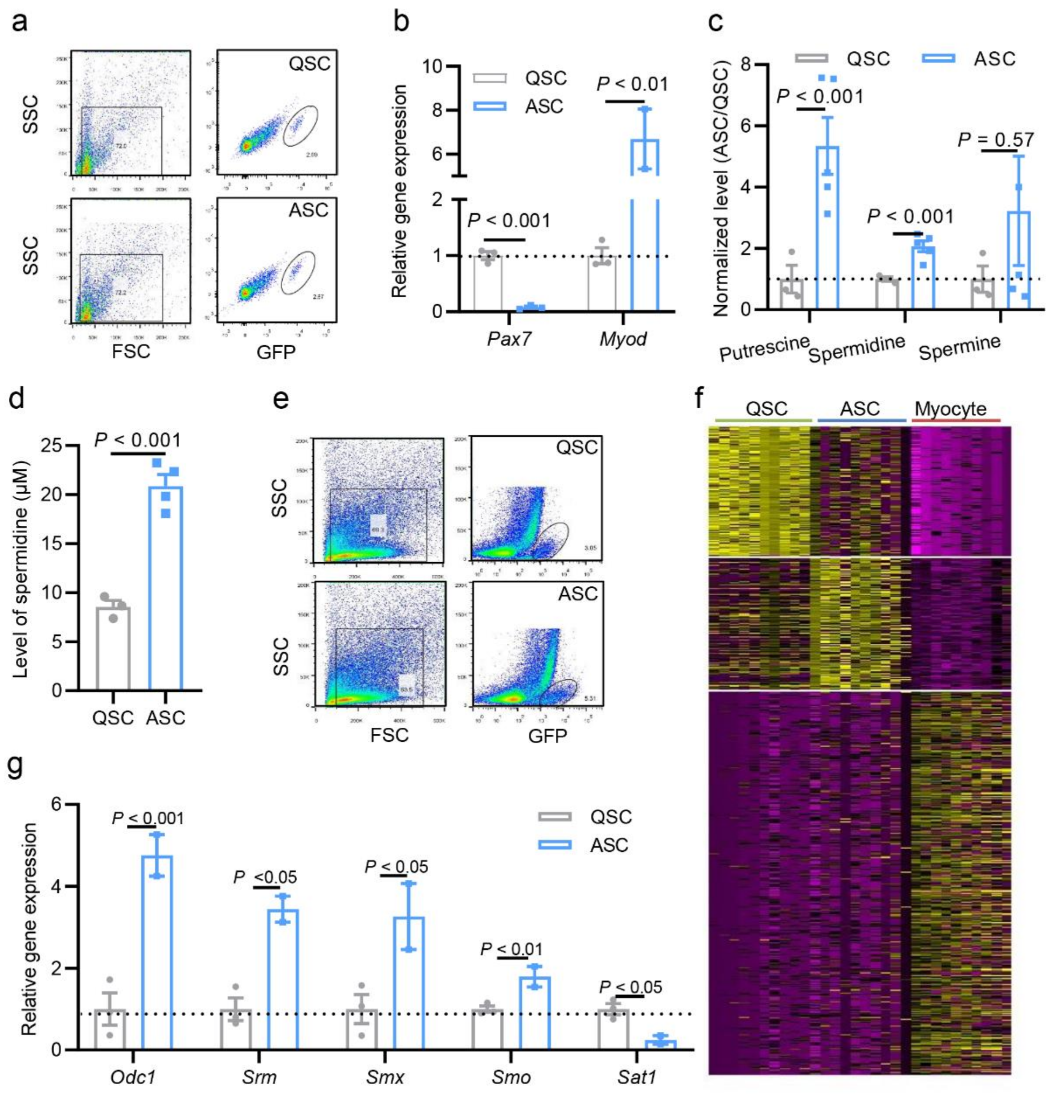

Supplementary Fig. S1. Polyamine levels are significantly increased in activated SCs. a Gating strategy for fluorescence-activated cell sorting (FACS) of QSCs and ASCs from TA muscles of 8-week-old *Pax7-nGFP* mice. **b** Relative expression levels of *Pax7* and *Myod* in FACS-sorted QSCs (n = 3) and ASCs (n = 3), as determined by RT-qPCR. Mean ± s.e.m. Two-way ANOVA. **c** Relative levels of the indicated polyamine species in ASCs (n = 5) normalized to that in QSCs (n = 3), as determined by the targeted metabolomics described in **Fig. 1a**. Mean ± s.e.m. Two-way ANOVA. **d** Absolute levels of spermidine in QSCs (n =3) and ASCs (n = 5). Mean ± s.e.m. Two-tail Student's t-test. **e** Gating strategy for FACS of QSCs and ASCs from TA muscles of 8-week-old *Pax7*$^{\text{CreERT2/+}}$*;Rosa26*$^{\text{mTmG/+}}$ mice. **f** Heatmap showing differentially expressed genes between QSCs and ASCs, as determined by the scRNA-seq analysis described in **Fig. 1e**. **g** Relative expression levels of the indicated genes in QSCs and ASCs, as determined

by bulk cell RNA-seq[4]. Mean ± s.e.m. Two-way ANOVA.

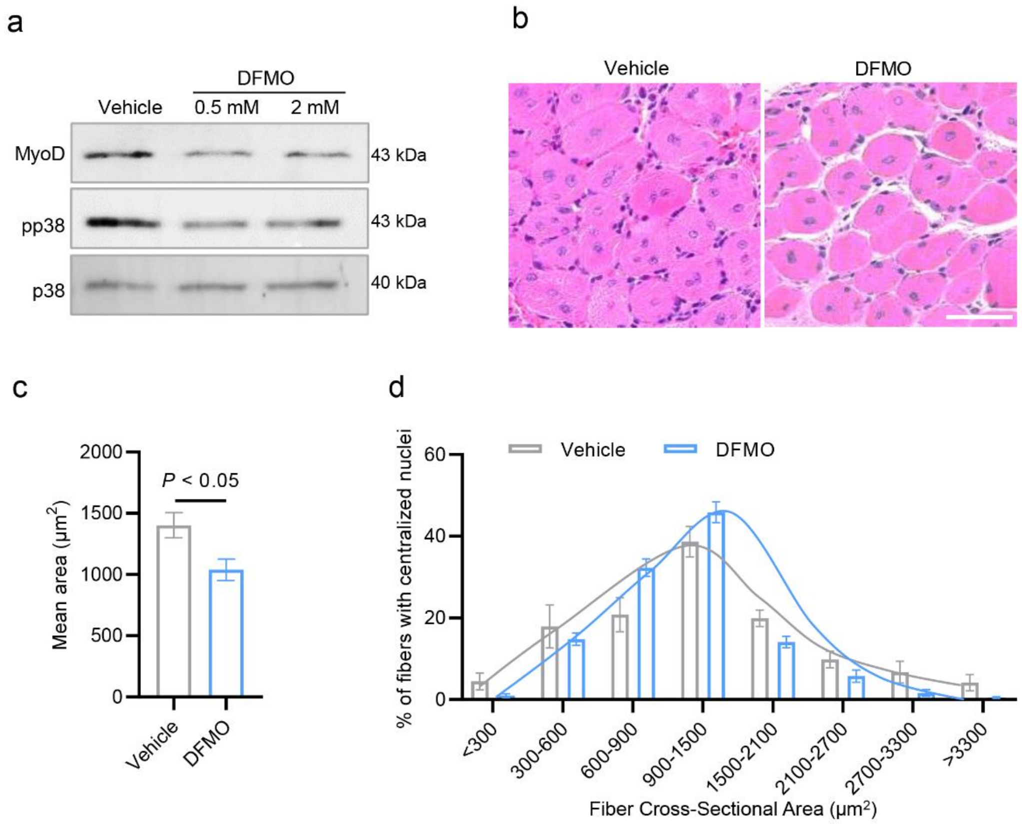

Supplementary Fig. S2. Blocking polyamine biosynthesis significantly suppresses SCs activation and skeletal muscle regeneration. a Western blots showing protein levels of MyoD, phosphorylated form of p38 and total p38 in FACS-sorted SCs treated with the indicated dose of DFMO for 6 h. PBS treatment served as vehicle control. **b** Representative images of hematoxylin and eosin (H&E)-stained cross-sections of the injured TA muscle with intramuscularly administration of DFMO, sampled at 7 days after the CTX-induced injury. Scale bars, 50 μm. **c** Mean cross-sectional area of regenerated myofibers with centralized nuclei, as measured from the H&E-stained cross-sections presented in **b**. *n* = 4 per group. Mean ± s.e.m. Two-tail Student's *t*-test. **d** Size distribution of the regenerated myofibers described in **c**. *n* = 4 per group.

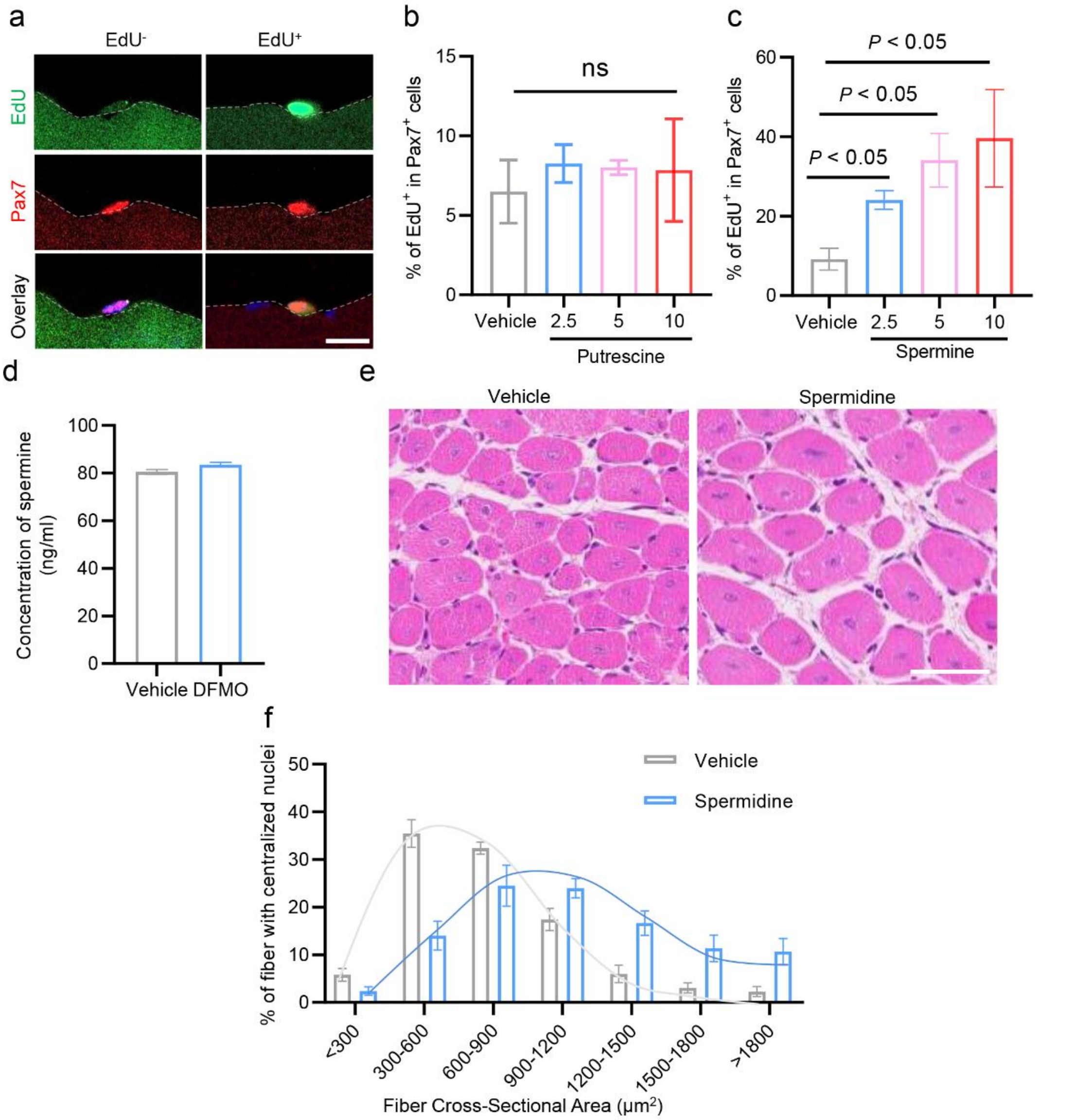

Supplementary Fig. S3. Spermidine is required for SC activation and muscle regeneration. **a** Representative immunostaining of Pax7 (red) and EdU (green) in single myofibers cultured *ex vivo* for 24 h. DAPI (blue) served to visualize nuclei. Scale bar, 20 μm. **b** Percentage of EdU^+ cells among total $Pax7^+$ cells in single myofibers treated with various doses of putrescine (2.5, 5, 10 μM) for 24 h. $n = 4$ for each group. Mean ± s.e.m. One-way ANOVA. **c** Percentage of EdU^+ cells among total $Pax7^+$ cells in single myofibers treated with various doses of spermine (2.5, 5, 10 μM) for 24 h. $n = 4$ for each group. Mean ± s.e.m. One-way ANOVA. **d** Spermine levels in FACS-sorted SCs cultured in presence of 0.5 mM of DFMO for 24 h, measured by LC-MS methods. **e** Representative images of H&E-stained cross-sections of the injured TA muscle with intramuscularly administration of spermidine, sampled at 7 days after the CTX-

induced injury. Scale bars, 50 μm. **f** Cross-sectional area of regenerated myofibers with centralized nuclei, as measured from the H&E-stained cross-sections presented in **e**. *n* = 4 per group. Mean ± s.e.m.

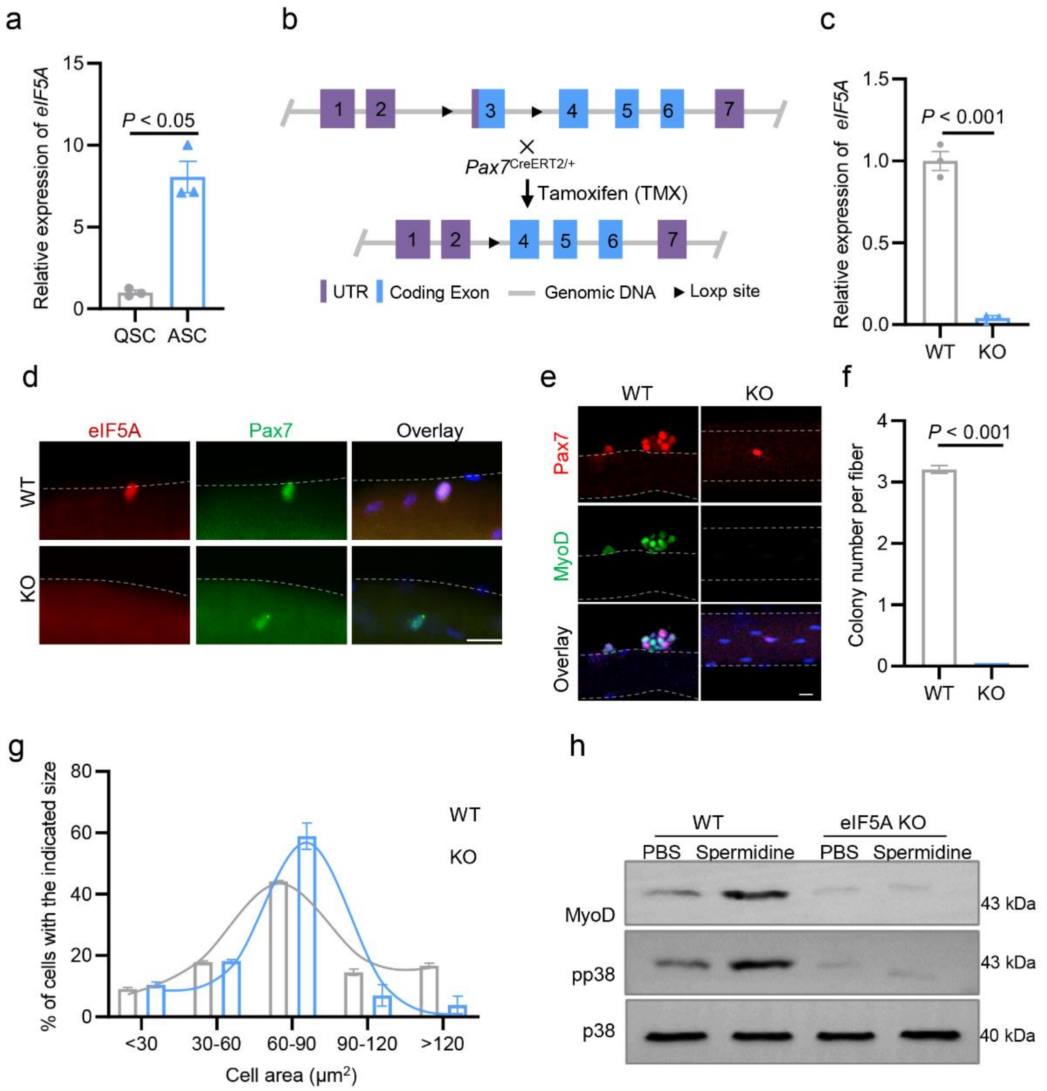

Supplementary Fig. S4. eIF5A is essential for SC activation and skeletal muscle regeneration. a Relative expression of *eIF5A* in QSCs and ASCs, as determined by RT-qPCR. $n = 3$ per group. Mean ± s.e.m. Two-tail Student's *t*-test. **b** Schematic diagram showing generation of inducible SC-specific *eIF5A*-knockout (KO) mice ($Pax7^{CreERT2/+}$;$eIF5A^{f/f}$) and WT controls ($Pax7^{CreERT2/+}$). **c** Relative expression levels of *eIF5A* in SCs freshly isolated from WT and KO mice. $n = 3$ per group. Mean ± s.e.m. Two-tail Student's *t*-test. **d** Representative immunostaining of eIF5A (red) and Pax7 (green) in EDL-derived single myofibers obtained from WT and KO mice and cultured *ex vivo* for 36 h. DAPI (blue) served to visualize nuclei. Scale bar, 20 μm. **e** Representative immunostaining of Pax7 (red) and MyoD (green) in EDL-derived single myofibers obtained from KO and WT mice and cultured *ex vivo* for 72 h. DAPI (blue) served to visualize nuclei. Scale bars, 20 μm. **f** Numbers of colonies per EDL myofiber from KO and WT mice, calculated based on the Pax7 and MyoD immunostaining described in

e. *n* = 4 for each group. Mean ± s.e.m. Two-tail Student's *t*-test. **g** Area of SCs obtained from WT and KO mice and cultured *in vitro* for 18 h. *n* = 3 for each group. **h** Western blots showing protein levels of MyoD, phosphorylated form of p38 and total p38 in FACS-sorted SCs isolated from WT and KO mice treated with spermidine for 6 h. PBS treatment served as vehicle control.

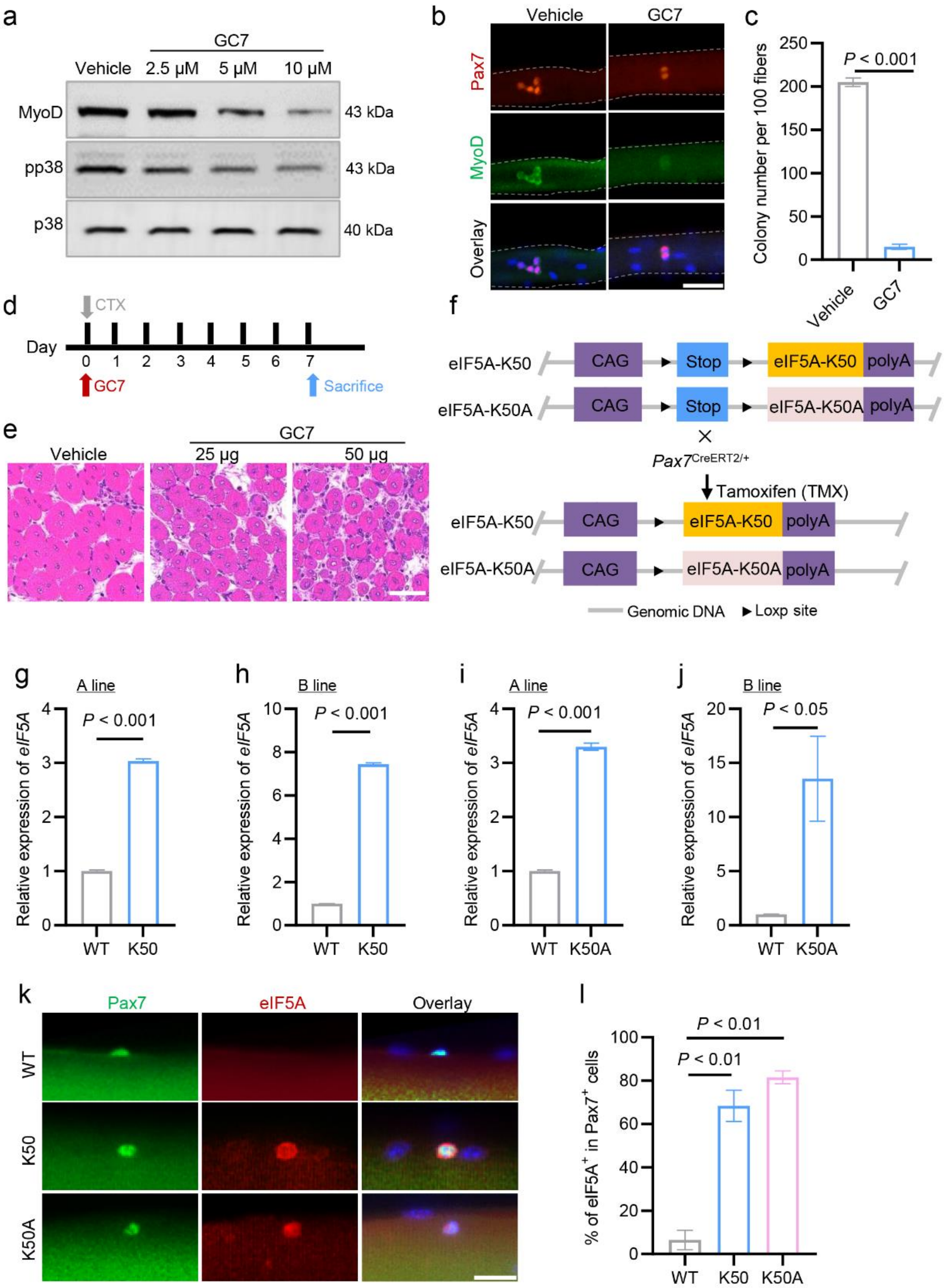

Supplementary Fig. S5. Spermidine stimulates SC activation by generating hypusinated eIF5A. a Western blots showing protein levels of MyoD, phosphorylated form of p38 and total p38 in FACS-sorted SCs treated with the indicated dose of GC7 for 6 h. **b** Representative SC colonies visualized by immunostaining of Pax7 (red) and MyoD (green) in EDL-derived single myofibers obtained from C57BL/6j mice and cultured ex vivo in the presence or absence of the inhibitor, GC7 (10 μM), for 72 h. DAPI (blue) served to visualize nuclei. Scale bar, 50 μm. **c**

Colony numbers per 100 of the myofibers described in d. n = 4 for each group. Mean ± s.e.m. Two-tail Student's t-test. **d** Experimental scheme for CTX-induced muscle injury and regeneration in the presence of GC7 (0, 25, 50 μg). **e** Representative H&E-stained cross-sections of TA muscles obtained from C57BL/6j mice on day 7 after CTX-induced injury and treated with various dose of the inhibitor, GC7 (0, 25, 50 μg). Scale bar, 50 μm. **f** Schematic diagram showing generation of *eIF5A*-TG mice and mutant *eIF5A* (K50A)-TG mice. **g, h** Relative expression of *eIF5A* in SC isolated from *eIF5A-K50* transgenic mice. Two lines of *eIF5A-K50* transgenic mice (A and B) were used. The data were determined by RT-qPCR and presented as mean ± s.e.m from three mice for each genotype. *n* = 3. Two tail Student's *t*-test. **i, j** Relative expression of *eIF5A* in SC isolated from *eIF5A-K50A* transgenic mice. Two lines of *eIF5A-K50A* transgenic mice (A and B) were used. The data were determined by RT-qPCR and presented as mean ± s.e.m from three mice for each genotype. *n* = 3. Two tail Student's *t*-test. **k** Representative immunostaining of Pax7 (green) and eIF5A (red) in freshly isolated single myofibers obtained from EDL muscles of WT, TG-K50, and mutTG-K50A mice. DAPI (blue) served to visualize nuclei. Scale bar, 20 μm. **l** Percentage of $eIF5A^+$ cells among total $Pax7^+$ cells in the single myofibers described in **k**. *n* = 4 for each group. Mean ± s.e.m. One-way ANOVA.

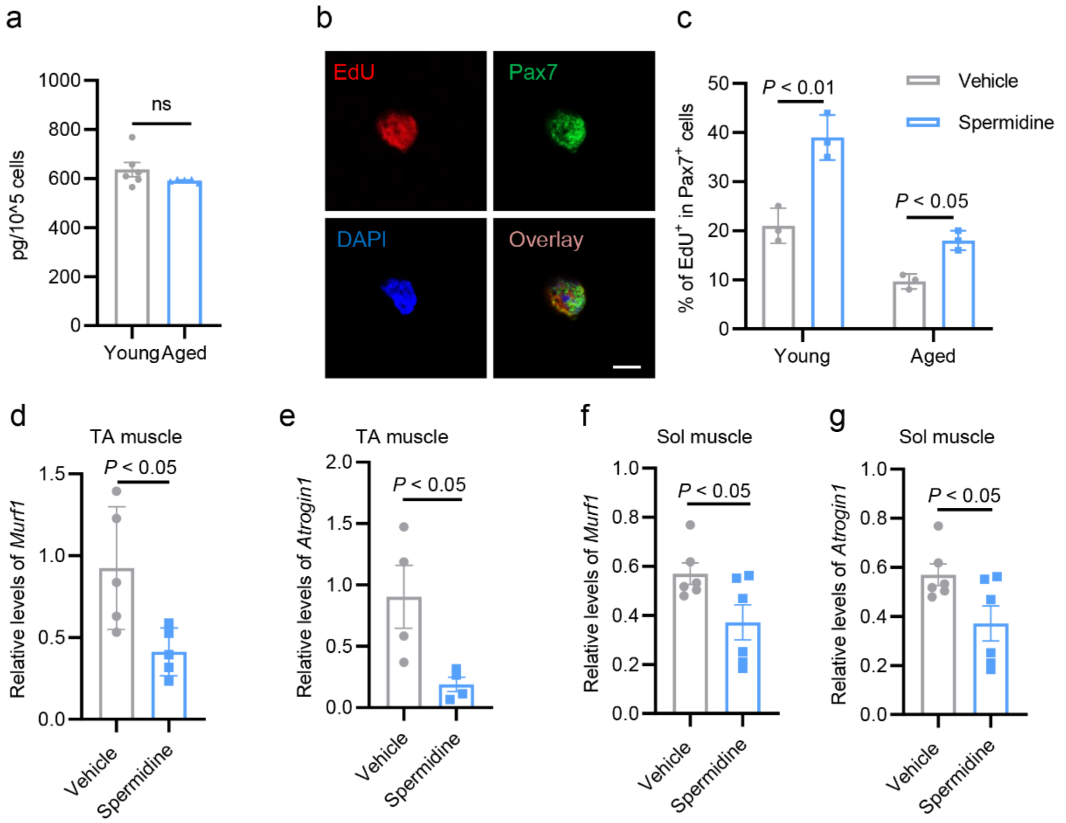

Supplementary Fig. S6. Spermidine promotes activation of aged SCs and improves muscle physiological function in aged mice. a Level of spermine in SCs isolated from young (6 months) and aged (30 months) mice. $n = 5$ for each group. Mean ± s.e.m. Two-tail Student's *t*-test. **b** Representative images of immunostaining for Pax7 (green) and EdU (red) among FACS-resolved SCs that had been cultured *in vitro* for 36 h. DAPI (blue) served to visualize nuclei. **c** Percentage of EdU^+ cells among total $Pax7^+$ cells of SCs isolated from young and aged mice and treated with 10 µM of spermidine for 36 h. PBS served as a control (vehicle). $n = 3$ per group. Mean ± s.e.m. Two-way ANOVA. **d,f** Relative levels of *Murf-1* in *Tibialis Anterior* (TA) muscles (**d**) and *Soleus* (Sol) muscles (**f**) from the aged mice described in **Fig. 6f**, as determined by RT-qPCR. $n = 5$ per group. Mean ± s.e.m. Two-tail Student's *t*-test. **e,g** Relative levels of *Atrogin-1* in *Tibialis Anterior* (TA) muscles (**e**) and *Soleus* (Sol) muscles (**g**) from the aged mice described in **Fig. 6f**, as determined by RT-qPCR. $n = 5$ per group. Mean ± s.e.m. Two-tail Student's *t*-test.

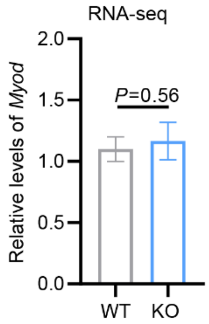

Supplementary Fig. S7. eIF5A selectively translates *Myod* mRNA in ASCs. Relative levels of *Myod* in *eIF5A*-KO and WT cells, as determined by triplicate experiments of bulk cell RNA-seq. Mean ± s.e.m. Two-tail Student's *t*-test.

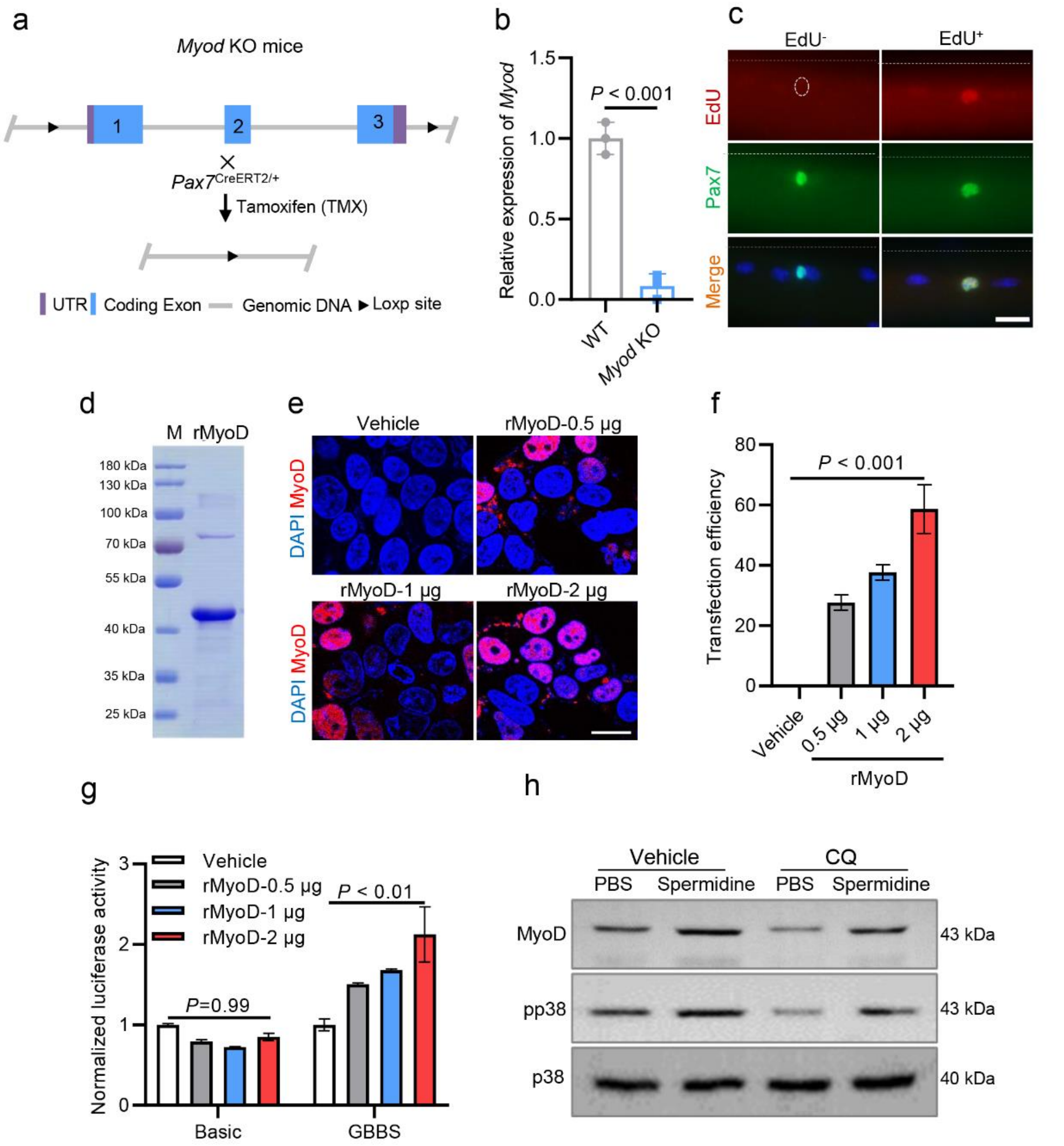

Supplementary Fig. S8. Spermidine-eIF5A-mediated translation of *Myod* is required for SC activation. a Schematic diagram showing generation of inducible SC-specific *Myod*-KO mice (*Pax7*$^{\mathrm{CreERT2/+}}$;*Myod*$^{\mathrm{f/f}}$). **b** Relative expression levels of *Myod* in SCs from *Myod*-KO mice and WT controls, as determined by RT-qPCR. $n = 3$ per group. Mean ± s.e.m. Two-tail Student's *t*-test. **c** Representative images of EdU (red) and Pax7 (green) in EDL-derived single myofibers obtained from inducible SC-specific *Myod*-KO mice and WT controls. DAPI served to visualize nuclei. Scale bar, 20 μm. **d** Coomassie brilliant blue-stained SDS-PAGE gel showing recombinant His-tagged-MyoD protein. **e** Representative images of immunofluorescent staining of MyoD (red) in HEK293 cells tranfected with the indicated dose of MyoD. DAPI (blue) served to visualize nuclei. Scale bar, 50 μm. **f** Transfection efficiency were quantified in

e. **g** Luciferase activity assay in HEK293 cells co-transfected with the plasmids of MyoG promoter reporter and various dose of MyoD protein. GBBS is a plasmid containing MyoG gene promoter. Basic is a plasmid without any promoter elements. Data are presented as mean ± s.e.m from three independent experiments. Significance was assessed by Two-way ANOVA. **h** Western blots showing protein levels of MyoD, phosphorylated form of p38 (pp38) and total p38 in FACS-sorted MuSCs treated with spermidine in presecnce or absence CQ for 6 h. DMSO treatment as control (vehicle).
